# Global Genome Analysis of the Downstream Binding Targets of Testis Determining Factor SRY and SOX9

**DOI:** 10.1371/journal.pone.0043380

**Published:** 2012-09-12

**Authors:** Ramji K. Bhandari, Md. M. Haque, Michael K. Skinner

**Affiliations:** Center for Reproductive Biology, School of Biological Sciences, Washington State University, Pullman, Washington, United States of America; Massachusetts General Hospital, United States of America

## Abstract

A major event in mammalian male sex determination is the induction of the testis determining factor *Sry* and its downstream gene *Sox9*. The current study provides one of the first genome wide analyses of the downstream gene binding targets for SRY and SOX9 to help elucidate the molecular control of Sertoli cell differentiation and testis development. A modified ChIP-Chip analysis using a comparative hybridization was used to identify 71 direct downstream binding targets for SRY and 109 binding targets for SOX9. Interestingly, only 5 gene targets overlapped between SRY and SOX9. In addition to the direct response element binding gene targets, a large number of atypical binding gene targets were identified for both SRY and SOX9. Bioinformatic analysis of the downstream binding targets identified gene networks and cellular pathways potentially involved in the induction of Sertoli cell differentiation and testis development. The specific DNA sequence binding site motifs for both SRY and SOX9 were identified. Observations provide insights into the molecular control of male gonadal sex determination.

## Introduction

The process of mammalian sex determination was described by Alfred Jost in the 1940's as 1) chromosomal, 2) gonadal and 3) sexual differentiation [1], [2], [3]. The chromosomal composition determines the genotypic sex (XY as males and XX as females) at fertilization. Mammalian embryos are considered sexually indifferent until the transient action of a Y-linked testis determining factor (TDF) that initiates gonadal differentiation into the testis [4]. Although a number of candidate TDF genes were suggested [5], [6], [7], it was not until 1990 that the sex determining region of the Y-chromosome (SRY) was identified to induce Sertoli cell differentiation and testis development [8], [9]. After finding *Sry* as a master male sex determining gene it was hypothesized that testis development must involve SRY interactions with other autosomal genes involved in the regulation of Sertoli cell differentiation as downstream targets of SRY [10]. Testis determination in the mouse is initiated at embryonic day E10.5 after the *Sry* is expressed in precursor Sertoli cells of the indifferent gonad. SRY up-regulates the expression of an autosomal related HMG-box gene, *Sox9*, that promotes the further differentiation of Sertoli cells [11], [12]. An example of a SOX9 downstream target is Anti-Müllerian Hormone (AMH) [13], [14]. The expression of *Sox9* is potentiated by actions of fibroblast growth factor (FGF9) and prostaglandin D2 in Sertoli cells [15], [16], [17]. SOX9 reaches a critical threshold that represses *Sry* expression via a SOX9-dependent negative feedback loop [11], [18]. It is believed that at this stage SOX9 functionally replaces SRY and acts downstream to further promote the differentiation of the Sertoli cell in the fetal testis. Genetic mutational studies suggest that the loss of function of SRY and SOX9 in XY embryos results in male-to-female sex reversal [9], [10]. In contrast, as the gain of function in XX embryos induces male development [10], indicating that these two genes cooperate in testis determination [19], [20], [21], [22], [23]. The current study was designed to use a genome wide analysis to identify additional downstream targets of SRY and SOX9 to further elucidate the molecular control mechanism of mammalian male sex determination.

Testis differentiation involves the actions of a genome wide network of genes initiated by SRY. In contrast, ovary differentiation has been thought to be passive and initiated in the absence of SRY expression. The complex biological process of either testis or ovary development requires active networks of factors that tip the balance of phenotypic sexes. For example, SOX9, FGF9, PDGS, DMRT1 promote testis differentiation, while factors such as WNT4, FOXL2, RSPO1 repress male genes to promote ovary development [22]. These mutually antagonistic forces lead to the development of opposite gonadal sex. However, in a systems biology perspective it is likely that genome wide networks of the genes that regulate this critical biological process will be required and not a few selected genes.

Currently it is widely believed that the primary function of SRY is to trigger molecular events underlying fetal testis differentiation through the induction of *Sox9* expression. At the time of Sertoli cell differentiation and testis development, SRY and steroidogenic factor 1 (SF1) synergistically act on testis-specific enhancer region of the *Sox9* promoter to induce testis-specific expression of *Sox9*. Therefore, *Sox9* has been found to be one of the direct downstream targets of the protein encoding SRY [24]. In 2009, cerebillin precursor 4 (*Cbln4*) gene was found to be one of the downstream targets of SRY and SOX9 [25]. Cbln4 encodes a transmembrane protein and is expressed in a male-specific manner, but the function of Cbln4 product in testicular development is not known. Recently, we have shown in the rat that the basic helix-loop-helix transcription factor *Tcf21* and the growth factor neurotrophin 3 (*Ntf3*) are direct downstream targets of SRY [26], [27]. TCF21 and NTF3 have previously been reported to have roles in formation of testis cords [28], [29]. Therefore, the majority of SRY downstream genes remain to be elucidated.

Following the expression of *Sry* in precursor Sertoli cells of the bipotential gonad a myriad of genes are thought to act in networks to initiate the differentiation of precursor cells into Sertoli cells. In the absence of SRY, testis differentiation derails and leads to the development of an ovary. Mutually exclusive and equally powerful molecular forces are acting to ensure the differentiation of the bipotential gonad into proper gonadal structures [19]. Interestingly, even in the presence of SRY the conditional deletion of Sox9 in precursor Sertoli cells causes a distinct male-to-female sex reversal with a multitude of molecular changes, suggesting that SRY and SOX9 have common functions but specific molecular targets [30]. Studies over the past two decades have expanded our understanding of the importance of SRY and SOX9 in testis differentiation, however, the information on how these two determinants are acting downstream to promote testis differentiation is not known. The current study took an *in vivo* genome wide systems level approach to pull down downstream binding targets from rat embryonic gonads that were undergoing male sex differentiation. Observations identify the direct downstream binding targets of SRY and SOX9. Global gene networks of binding targets of these two critical sex determination factors provides insight and opportunities for future investigation of the molecular control of Sertoli cell differentiation and testis development.

## Results

### Downstream Binding Targets of SRY

Since the discovery of SRY as a male sex determination and testis determining factor [8], [9], only a few genes have been identified as *in vivo* direct downstream targets. Currently, only *Sox9, Tcf21, Ntf3* and *Cbln4* are reported as downstream targets of SRY. The existence of other downstream targets has been debated [21], [22], [23]. One of the limitations for not having any significant progress in finding downstream targets is the classic *in vivo* chromatin immunoprecipitation (ChIP) method that requires a large amount of chromatin for performing a ChIP assay and detection of low affinity binding sites. To perform a conventional cross-linked ChIP assay at least 5 million cells are required. During gonadal sex determination the gonads are small and precursor Sertoli cells that express *Sry* are low in number. In order to collect 5 million *Sry* expressing cells hundreds of embryos are required. To overcome this issue we established a native (not involving cross-linking) ChIP assay that utilizes twenty to thirty embryonic testis and carrier cell chromatin of fly origin. The native ChIP identifies the high affinity binding sites and reduces lower affinity sites compared to the conventional ChIP. The protocol developed involved the use of genome wide promoter tiling arrays to perform a ChIP-Chip assay using a competitive hybridization with non-immune IgG to eliminate false positives. The cell purity of the samples is irrelevant since the only site of SRY and SOX9 is the Sertoli cell in the gonad. The SRY or SOX9 Chip will only pull-down the Sertoli cell targets. The method dramatically increased sensitivity and specificity of the analysis.

**Figure 1:**
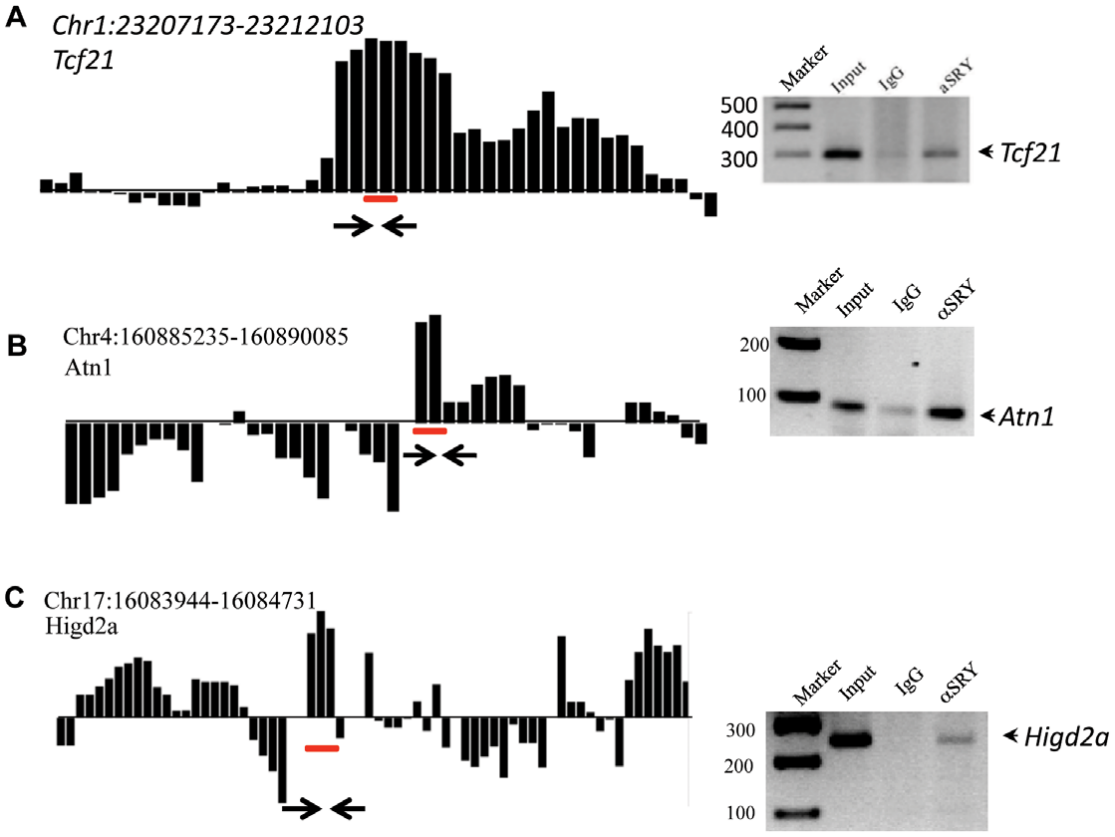
Representative examples SRY downstream direct binding target gene promoters for (A) *Tcf21*, (B) *Atn1*, and (C) *Higd2a*. The positive hybridization is specific to SRY ChIP-DNA signal and negative hybridization to the non-immune IgG ChIP-DNA signal. Hybridization signals are the average of three biological replicates of ChIP assays. Hybridization signals below the statistical significance of p<1×10^−7^ was not considered. The localization of SRY response element motif is indicated for each promoter as a horizontal line under the bar. PCR primers were designed from the position indicated by two arrows. The PCR gel identifies PCR product size with (Markers), genomic DNA (Input), IgG ChIP (IgG) and SRY ChIP (aSRY). Data represent ChIP-PCR assays from three different experiments and biological replicates.

Initially this ChIP approach was used to identify *Tcf21* and *Ntf3* as direct downstream SRY target genes [26], [27]. In order to pull-down all the downstream targets three independent ChIP assays with a specific SRY antibody on chromatin from E13 rat testis (13–18 tail somite stage embryos) were performed. A comparative hybridization of SRY-ChIP DNA with non-immune IgG-ChIP DNA to a rat promoter tiling array (Nimblegen) containing at least 4–5 kb for each of 15,287 proximal-promoter regions in the rat genome was performed. To identify genes bound by SRY and SOX9 a sliding window of 600 bp (the majority of our ChIP DNA fragments ranged approximately 400–600 bp) was used to detect SRY- specific enrichment present in proximal-promoter regions. This required the detection of at least three consecutive probes in all three replicates at a statistical significance level less than p<0.001. A total of 1773 promoters were represented. To select the target promoters from this analysis a cut-off of p<1×10^−7^ was used that identified 71 genes as direct downstream binding targets with distinct SRY binding sites (response elements, [T/A]AACAA[T/A] & [T/A]TTGTT[A/T]) (Table 1). Atypical binding targets without any SRY binding site identified 159 additional promoters (Supplementary Table S1). A representative example of downstream direct targets is shown in Figure 1. PCR confirmation of each representative SRY site for the downstream binding target is shown next to the ChIP-Chip hybridization plot.

**Table 1:** Direct downstream binding targets of SRY during male sex determination.

Gene Symbol and Category	GenBank/Reference Sequence	Binding Site Chromosomal Location	p-value	# of SRY Motifs	Gene Title
**Apoptosis**					
Higd2a	NM_001106102	chr17:16083944-16084731	2.16E-21	1	HIG1 hypoxia inducible domain family, member 2A
Pdcd6ip	NM_001029910	chr8:118351413-118352013	1.59E-21	1	Programmed cell death 6 interacting protein
**Cell Cycle**					
RGD1560888	NM_001109061	chr8:40571831-40572431	5.07E-08	1	Similar to Cell division protein kinase 8
**Development**					
Atn1	NM_017228	chr4:160887462-160888360	1.28E-12	1	Atrophin 1
Crygb	NM_001109875	chr9:63736296-63737096	2.92E-11	1	Crystallin, gamma B
Lrrc68	NM_001107482	chr1:78888269-78888869	1.59E-08	2	Leucine rich repeat containing 68
Lcn11	NM_001135809	chr3:3888687-3889792	8.46E-09	1	Lipocalin 11
RGD1565947	NM_001106780	chr7:30717183-30717983	2.25E-09	3	Similar to netrin 4
Sar1b	NM_001009622	chr10:37281198-37281897	4.60E-10	3	SAR1 homolog B (S. cerevisiae)
Tex10	NM_001106653	chr5:65166327-65167127	4.78E-09	1	Testis expressed 10
**Electron Transport**					
Pdcl3	NM_001025709	chr9:38002255-38002855	8.65E-09	3	Phosducin-like 3
**Immune Response**					
Cd24	NM_012752	chr20:47499009-47499609	2.95E-20	2	CD24 molecule
Sec1	NM_001135584	chr1:96149778-96150598	4.18E-08	1	Secretory blood group 1
**Metabolism & Transport**					
Afg3l2	NM_001134864	chr18:63975093-63975767	6.73E-16	2	AFG3(ATPase family gene 3)-like 2
Exoc4	NM_053875	chr4:60405518-60406118	8.64E-09	1	Exocyst complex component 4
Gltp	NM_001134413	chr12:43194112-43195012	8.14E-32	1	Glycolipid transfer protein
Hpgds	NM_031644	chr4:94639300-94639900	2.34E-09	1	hematopoietic prostaglandin D synthase
Hyal1	NM_207616	chr8:112828762-112829362	4.39E-14	1	Hyaluronoglucosaminidase 1
Hyal3	NM_207599	chr8:112828762-112829362	4.39E-14	1	Hyaluronoglucosaminidase 3
Sdha	NM_130428	chr1:29738109-29738709	1.53E-10	1	Succinate dehydrogenase complex, A
Sdhd	NM_198788	chr8:53967070-53967670	4.44E-08	1	Succinate dehydrogenase complex, subunit D
Sec24a	NM_001105780	chr10:37281198-37281897	4.60E-10	3	SEC24 family, member A (S. cerevisiae)
Timm8b	NM_022541	chr8:53967070-53967670	4.44E-08	1	Translocase of inner mitochondrial membrane 8
Tmed4	NM_001107238	chr14:87002671-87003360	5.63E-08	1	Transmembrane emp24 protein transport 4
Tpi1	NM_022922	chr4:160936834-160937434	4.38E-11	1	Triosephosphate isomerase 1
**Proteolysis**					
Cpa2	NM_001013083	chr4:57449368-57449968	6.76E-08	1	Carboxypeptidase A2 (pancreatic)
Cul2	NM_001108417	chr17:62742775-62743470	1.46E-08	2	Cullin 2
LOC689226	BC167074	chr7:18614322-18614922	9.65E-11	2	Similar to ubiquitin-conjugating enzyme E2R 2
Tmprss6	NM_001130556	chr7:116422114-116422899	2.30E-12	1	Transmembrane protease, serine 6
**Receptors & Binding Proteins**					
Grm2	NM_001105711	chr8:111851299-111852092	5.01E-09	2	Glutamate receptor, metabotropic 2
Il1rapl1	NM_177935	chrX:74472070-74472768	2.96E-14	4	interleukin 1 receptor accessory protein-like 1
Olr122	NM_001000156	chr1:161468724-161469324	4.12E-08	3	Olfactory receptor 122
Olr1553	NM_001000051	chr11:42266451-42267051	1.33E-10	1	Olfactory receptor 553
Olr1657	NM_001000536	chr17:50544822-50545500	2.98E-08	1	Olfactory receptor 1657
Olr463	NM_001000934	chr3:69098160-69098859	5.02E-08	1	Olfactory receptor 463
Olr669	NM_001000349	chr3:73187159-73187759	1.57E-08	2	Olfactory receptor 669
Olr770	NM_001000372	chr3:97220204-97221208	2.48E-09	1	Olfactory receptor 770
Olr853	NM_001000398	chr5:70398861-70399461	9.49E-08	1	Olfactory receptor 853
Vom1r59	AY510282	chr1:73592390-73592990	1.52E-10	1	vomeronasal 1 receptor, 59
Vom2r11	NM_001099470	chr1:57382648-57383341	7.39E-08	2	Vomeronasal 2 receptor 11
Vom2r8	NM_001099464	chr1:49588680-49589280	1.60E-08	2	Vomeronasal 21 receptor 8
**Signaling**					
Jkamp	NM_001106738	chr6:94284244-94284963	1.46E-23	1	JNK1/MAPK8-associated membrane protein
RGD1562638	NM_001100944	chr16:68750676-68751465	6.94E-22	1	MAP/microtubule affinity-regulating kinase 3
**Transcription**					
Armc7	BC166454	chr10:105554314-105555129	5.38E-08	3	armadillo repeat containing 7
Btbd4	NM_001107808	chr3:170570362-170570962	3.04E-09	1	Zinc finger and BTB domain containing 46
Ccdc127	NM_198766	chr1:29738109-29738709	1.53E-10	1	Coiled-coil domain containing 127
Gata1	NM_012764	chrX:26564683-26565464	7.55E-08	1	GATA binding protein 1
Maff	NM_001130573	chr7:117329082-117329787	3.55E-08	1	V-maf musculoaponeurotic fibrosarcoma F
Meis1	NM_001134702	chr14:99835529-99836129	4.65E-15	2	Meis homeobox 1
Nop16	NM_001047095	chr17:16083944-16084731	2.16E-21	1	NOP16 nucleolar protein homolog (yeast)
Nsfl1c	NM_031981	chr3:141798504-141799104	2.81E-08	2	NSFL1 (p97) cofactor (p47)
Phox2a	NM_053869	chr1:159272192-159273082	4.67E-14	1	Paired-like homeobox 2a
Rag1	NM_053468	chr3:86795336-86796015	1.34E-11	2	Recombination activating gene 1
Rai14	NM_001011947	chr2:60062344-60063029	1.37E-09	1	Retinoic acid induced 14
Zfp354a	NM_052798	chr10:36652409-36653009	1.28E-08	1	Zinc finger protein 354A
Znf507	NM_001106248	chr1:88364838-88365940	3.47E-12	4	Zinc finger protein 507
Ddx46	NM_139098	chr17:15030355-15030955	1.78E-13	3	DEAD (Asp-Glu-Ala-Asp) box polypeptide 46
Tcf21	NM_001032397	chr1:23209070-23210356	1.76E-10	3	Transcription factor 21/Pod1/Capsulin/Epicardin
**Translation & Protein Modification**					
Tsen2	NM_001014057	chr4:151669335-151669935	4.91E-08	1	tRNA splicing endonuclease 2 homolog
Arfgef2	NM_181083	chr3:157964936-157965536	1.30E-10	1	ADP-ribosylation factor guanine factor 2
Mrpl51	NM_001106621	chr4:161309657-161310257	4.88E-09	2	Mitochondrial ribosomal protein L51
Rpl24	NM_022515	chr11:45636727-45637615	1.07E-10	1	Ribosomal protein L24
Rtf1	NM_001108958	chr3:106189897-106190688	4.05E-09	1	Rtf1, Paf1/RNA polymerase II complex
**Miscellaneous & Unknown**					
Cytsa	NM_001039455	chr20:13946693-13947489	1.35E-09	1	Cytospin A
Dcaf7	NM_001107057	chr10:95425513-95426318	6.60E-12	1	DDB1 and CUL4 associated factor 7
Fam12b	NM_178103	chr15:27107548-27108246	1.48E-11	1	Epididymal protein 3B
LOC308990	NM_001025001	chr1:186339810-186340908	7.02E-10	3	Hypothetical protein LOC308990
RGD1303127	NM_001004244	chr7:137280915-137281694	3.89E-12	1	Similar to hypothetical protein FLJ20436
RGD1562533	BC127538	chr12:7302557-7303157	1.79E-08	1	Similar to mKIAA0774 protein
**ESTs**					
RGD1305721	NM_001108031	chr6:94284244-94284963	1.46E-23	1	Similar to RIKEN cDNA 2810055F11
LOC298139	NM_001013930	chr5:91664056-91664656	1.57E-09	1	Similar to RIKEN cDNA 2310003M01
RGD1307325	BC082052	chr16:49407004-49407724	6.84E-08	1	Similar to RIKEN cDNA 4933411K20

**Figure 2:**
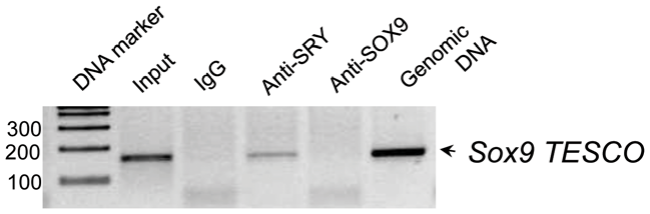
ChIP-PCR confirmation of *Sox9* as a downstream direct target of SRY. *Sox9* hybridization signals were not detected as the binding of SRY to Sox9 promoter is shown to occur at the TESCO region located at −7K upstream of the transcription start site. Non immune IgG was used as a negative control for each assay. ChIP DNA from IgG represented negative control throughout the experiment. PCR was conducted on 200 ng DNA amplified by whole genome amplification kit (Sigma). Data represent ChIP-PCR assay from three different experiments and biological replicates.

The three examples of SRY downstream direct targets shown are *Tcf21, Atn1*, and *Higd29*. The positive hybridization is specific to SRY-ChIP DNA hybridization and negative hybridization is specific to the non-immune IgG-ChIP-DNA, Figure 1(A–C). The localization of the SRY response element motif is indicated for each promoter. The other SRY downstream direct binding target ChIP-Chip hybridization profiles are shown in Supplemental Figure S1. Although *Sox9* is a direct downstream target of SRY, it was not detected by the ChIP-Chip assay. *Sox9* could not be found because hybridization probe sets contained only 4K base of the promoter regions and SRY binding to the *Sox9* promoter occurs at an upstream −7K base region of the promoter [24]. Therefore, the SRY-ChIP DNA was used to test the presence of *Sox9* by PCR in all three of the SRY-ChIP biological replicates, Figure 2. As expected, the SOX9 TESCO [23] binding site was detected in the SRY-ChIP DNA samples. Therefore, the two previously identified SRY targets SOX9 and TCF21 were detected in the SRY ChIP-Chip analysis helping validate the protocol. However, a limitation of the current study is that SRY binding sites outside of 4K base of the promoter would not be detected. This suggests the downstream binding targets detected will be a subset of a potentially larger set of SRY direct binding gene targets.

As a negative control, a non-immune IgG was used for each replicate of the ChIP assay. A comparative hybridization in the ChIP-Chip assay was then used to assess non-specific IgG binding. Hybridization results show that IgG also pulls down a significant number of gene promoter sites. In order to reduce the inclusion of false positives or false negatives in the list, manual screening of both SRY-bound and IgG-bound promoters at p<1×10^−7^ significance was performed. From 213 total IgG binding sites detected, 32 were found to be SRY binding positives, but their peaks were masked by adjacent larger peaks of IgG binding (Supplemental Figure S2, Supplemental Table S2). These binding target gene promoters were termed questionable positives and several were tested with PCR and found to be positive with SRY ChIP-PCR.

In addition to the direct SRY gene targets that contain an SRY response element motif, 159 atypical binding targets were identified that did not contain an SRY binding motif, Supplemental Table S1, but were identified in the ChIP-Chip assay. This suggests SRY associates with transcription factor complexes independent of DNA binding. Therefore, a large number of SRY binding sites are atypical downstream targets not involving DNA binding. An example of such an SRY indirect target was *Cbln4* that was previously shown to be downstream of SRY [25], but does not have an SRY binding sequence motif in the 4K base proximal promoter. The functional significance and mechanism of SRY regulation of these regions in the absence of SRY DNA sequence binding remains to be investigated.

### Downstream Binding Targets of SOX9

**Figure 3:**
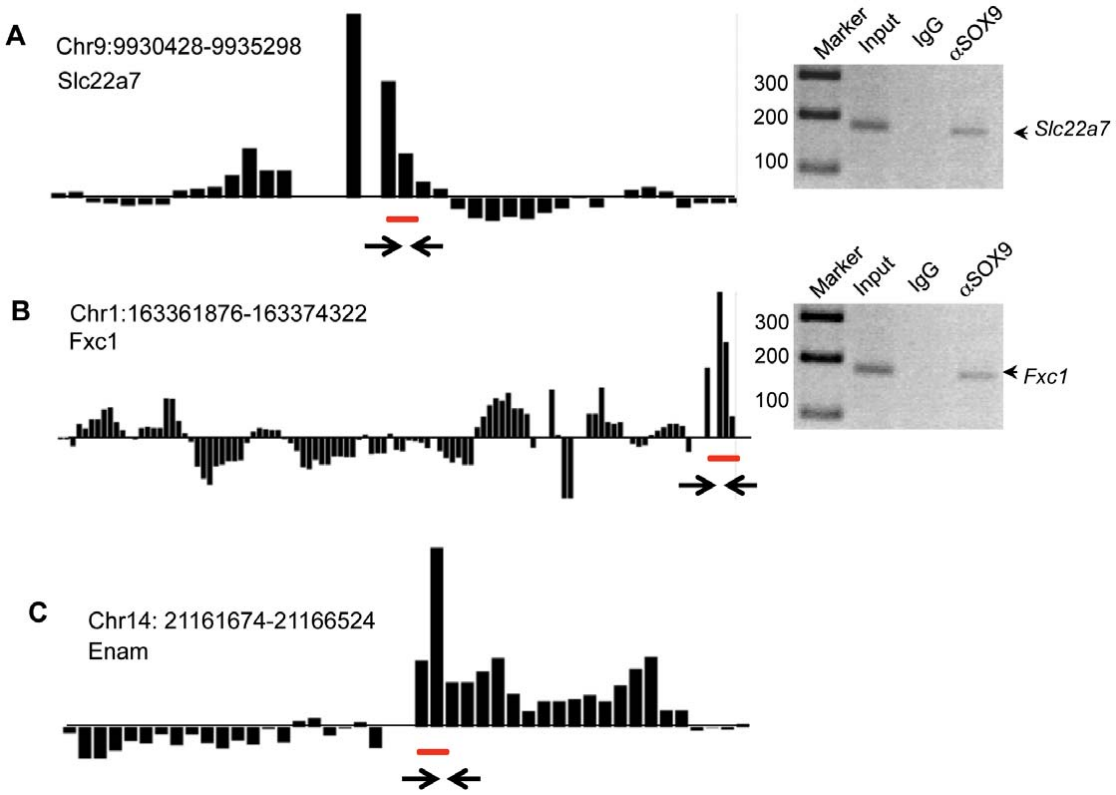
Representative examples of SOX9 binding to the target promoter. (A) *Slc22a7*, (B) *Fxc1*, and (C) *Enam*. The positive hybridization is specific to SOX9 ChIP-DNA signal (bar above the horizontal line) and negative hybridization (bar below the horizontal line) to the non-immune IgG ChIP-DNA signal. Hybridization signals are the average of three biological replicates of ChIP assays. Hybridization signals below the statistical significance of p<1×10^−7^ was not considered. The localization of SRY response element motif is indicated for each promoter as a horizontal line under the bar. PCR Primers were designed from the position indicated by two arrows. The PCR gel identifies PCR product size with (Markers), genomic DNA (Input), IgG ChIP (IgG) and SRY ChIP (aSRY). Data represent ChIP-PCR assays for three different experiments and biological replicates.

Downstream binding targets of SOX9 were analyzed using the same strategy as for SRY with an antibody specific for rat SOX9 [27]. Two *in vitro* confirmed distinct binding motifs [17], [31] were found for SOX9 binding: [T/C]TTG[T/A]G and [T/A]AACAA[T/A]. At a statistical significance of p<1×10^−7^, a total of 109 promoters were found to be direct binding targets of SOX9, Table 2. From these, 86 promoters have a SOX9 specific DNA sequence binding motif and 72 have an SRY HMG box DNA sequence binding motif. Combined there were 49 promoter regions with both SOX9 specific and HMG box binding motifs (Table 2). There were 24 promoter regions without a SOX9 binding motif (Supplemental Table S3) and 39 regions (Supplemental Table S4) with adjacent IgG negative peaks (termed here as questionable positives). Representative examples of downstream direct binding targets are shown in Figure 3 and PCR confirmation of each representative is shown next to the ChIP-Chip hybridization plot. Interestingly, the number of direct SRY binding targets of 71 and atypical binding targets of 159 when compared to the 109 direct SOX9 binding targets and 24 atypical binding targets suggests SRY has a much larger role in binding indirectly to transcription factor complexes compared to SOX9 that predominately has direct binding targets.

**Table 2:** Direct downstream binding targets of SOX9 during male sex determination.

Gene Symbol and Category	GenBank/Reference Sequence	Binding Site Chromosomal Location	p-value	# of motifs		Gene Title
				Sox9	SRY	
**Apoptosis**						
Higd2a	NM_001106102	chr17:16084544-16085239	1.38E-13	1	0	HIG1 hypoxia inducible 2A
**Cytoskeleton-ECM**						
Cntn4	NM_053879	chr4:141196887-141197487	3.01E-13	0	1	Contactin 4
Dnah1	NM_001033655	chr16:6759427-6760027	1.94E-08	1	1	Dynein, axonemal, heavy chain 1
Enam	NM_001106001	chr14:21163829-21164930	1.11E-11	3	1	Enamelin
Fxc1	NM_053371	chr1:163372155-163373134	3.76E-13	3	1	Fractured callus expressed transcript 1
LOC689770	NM_001142304	chr4:165929907-165930589	1.75E-12	3	0	Similar to osteoclast inhibitory lectin
Pcdhb9	NM_001109390	chr18:30207966-30208648	2.85E-20	0	2	Protocadherin beta 9
Spast	NM_001108702	chr6:21051125-21051725	1.98E-10	2	0	Spastin
**Development**						
Ang1	NM_001006992	chr15:27080104-27080796	5.75E-30	0	1	Angiogenin, ribonuclease A family, member 1
Dscr3	NM_001108316	chr3:15864716-15865316	1.88E-10	2	1	Down syndrome critical region gene 3
Gsg1	NM_001013166	chr4:172210431-172211196	3.79E-08	1	0	Germ cell associated 1
Lmln	NM_001108843	chr11:69550989-69551670	3.81E-08	0	2	Leishmanolysin-like
Neurod6	NM_001109237	chr4:84479894-84480494	5.46E-08	1	0	Neurogenic differentiation 6
Per3	NM_023978	chr5:168190800-168191622	2.83E-12	3	1	Period homolog 3 (Drosophila)
Pcp4l1	NM_001126093	chr13:87085586-87086186	2.70E-08	1	0	Purkinje cell protein 4-like 1
Sv2b	NM_057207	chr1:130887128-130887728	8.05E-19	1	0	Synaptic vesicle glycoprotein 2b
**Electron Transport**						
Cyp11a1	NM_017286	chr8:61792218-61792818	2.97E-17	0	1	Cytochrome P450, family 11, a1
**Epigenetics**						
Wbscr22	NM_001135743	chr12:22726326-22726926	4.69E-10	3	1	Williams Beuren syndrome chromosome region 22
**Growth Factors**						
Ngfrap1	NM_053401	chrX:123585655-123586255	1.78E-10	1	0	Nerve growth factor receptor associated protein 1
**Immune Response**						
Hrg	NM_133428	chr11:80264162-80264838	1.74E-12	2	0	Hhistidine-rich glycoprotein
**Metabolism & Transport**						
Aox3l1	NM_001008522	chr9:56944915-56945610	5.72E-10	1	0	Aldehyde oxidase 3-like 1
Ehhadh	NM_133606	chr11:81472220-81472820	1.32E-14	1	1	Hydratase/3-hydroxyacyl CoA dehydrogenase
Exosc4	NM_001134860	chr7:114375307-114376102	1.22E-09	1	4	Exosome component 4
Gnpnat1	NM_001134756	chr15:21287426-21288103	2.56E-16	0	1	Glucosamine-phosphate N-acetyltransferase 1
Gsk3b	NM_032080	chr11:64429588-64430188	7.30E-08	1	0	Glycogen synthase kinase 3 beta
Hsd3b	M38179	chr2:193507626-193508226	4.87E-09	1	2	3 beta-hydroxysteroid dehydrogenase
Hsd3b1	NM_001007719	chr2:193507626-193508226	4.87E-09	1	2	hydroxy-delta-5-steroid dehydrogenase, 3 beta- and steroid delta-isomerase 1
LOC311352	NM_001014047	chr3:107941134-107941734	1.61E-08	2	0	Similar to Adenosine deaminase CG11994-PA
LOC500959	NM_001033072	chr8:24855879-24856479	3.90E-09	0	1	Similar to triosephosphate isomerase
Lypla1	NM_013006	chr5:14912620-14913220	1.85E-12	1	0	Lysophospholipase 1
Mblac2	NM_001108934	chr2:9807293-9807893	5.07E-30	2	6	Metallo-beta-lactamase domain containing 2
Ndufa10	NM_199495	chr6:62282076-62283066	4.34E-10	0	1	NADH dehydrogenase 1 alpha subcomplex 10
Pop1	NM_001130550	chr7:69940911-69941511	2.41E-23	2	0	processing of precursor 1
Slc22a7	NM_053537	chr9:9932589-9933384	5.04E-11	1	0	Solute carrier family 22, member 7
Ugt1a6	BC107931	chr9:87035929-87036629	8.29E-11	3	3	UDP glucuronosyltransferase 1 A6
Ugt1a7c	AF461738	chr9:87029719-87030319	9.85E-12	0	1	UDP glucuronosyltransferase 1 A7C
**Proteolysis**						
P22k15	NM_199266	chr3:138338313-138338913	1.71E-12	0	1	Cystatin related protein 2
Ube2s	NM_001106224	chr1:67742581-67743181	1.66E-11	1	2	Ubiquitin-conjugating enzyme E2S
**Receptors & Binding Proteins**						
Grm3	NM_001105712	chr4:20745927-20746648	1.17E-08	2	1	Glutamate receptor, metabotropic 3
Hspbp1	NM_139261	chr1:67879661-67880261	9.96E-19	1	0	HSPA (heat shock 70kDa) binding protein, cytoplasmic cochaperone 1
Ly49s3	AY747628	chr4:168209700-168210300	1.16E-17	1	1	Ly-49 stimulatory receptor 3
Olr1006	NM_001000075	chr7:6593954-6595067	1.12E-10	0	7	Olfactory receptor 1006
Olr1057	NM_001000072	chr7:7914900-7915500	9.52E-10	3	1	Olfactory receptor 1057
Olr1162	NM_001000870	chr8:18233917-18234517	4.36E-10	0	1	Olfactory receptor 1162
Olr1339	NM_001000481	chr8:43021887-43022487	5.95E-10	2	0	Olfactory receptor 1339
Olr1370	NM_001000979	chr10:12391699-12392299	2.70E-08	0	3	Olfactory receptor1370
Olr1496	NM_001000716	chr10:60966886-60967486	4.45E-08	1	3	Olfactory receptor 1496
Olr1533	NM_001000496	chr11:41855848-41856448	5.37E-12	0	1	Olfactory receptor 1533
Olr1546	NM_001001106	chr11:42154682-42155282	9.41E-08	2	2	Olfactory receptor 1546
Olr1622	NM_001000838	chr15:26338868-26339468	1.12E-11	1	1	Olfactory receptor 1622
Olr1737	NM_001001422	chr20:1384704-1385401	2.59E-08	4	2	Olfactory receptor 1737
Olr1738	NM_001006599	chr20:1384704-1385401	2.59E-08	4	2	Olfactory receptor 1738
Olr1742	NM_001001424	chr20:1436423-1437023	1.45E-08	2	1	Olfactory receptor 1742
Olr1766	NM_001000490	chrX:136392211-136392811	4.00E-10	0	1	Olfactory receptor 1766
Olr185	NM_001000183	chr1:162806198-162806895	6.91E-12	3	0	Olfactory Receptor 185
Olr186	NM_001001031	chr1:162806198-162806895	6.91E-12	3	0	Olfactory receptor 186
Olr331	NM_001000760	chr1:215223740-215224437	1.07E-13	2	2	Olfactory receptor 331
Olr340	NM_001000253	chr1:216001183-216001783	4.16E-10	1	0	Olfactory receptor 340
Olr522	NM_001000562	chr3:70113851-70114451	4.46E-08	0	2	Olfactory receptor 522
Olr557	NM_001000669	chr3:70965140-70965837	9.09E-08	0	2	Olfactory receptor 557
Olr621	NM_001000652	chr3:72132250-72133033	4.98E-23	3	3	Olfactory receptor 621
Olr664	NM_001000347	chr3:73090449-73091049	6.82E-09	2	4	Olfactory receptor 664
Olr734	NM_001000617	chr3:74546184-74546962	4.49E-08	2	3	Olfactory receptor 734
Olr770	NM_001000372	chr3:97220509-97221208	3.73E-16	2	1	Olfactory receptor 770
Olr795	NM_001000601	chr3:97991622-97992349	1.66E-08	1	0	Olfactory receptor 795
Olr81	NM_001001271	chr1:160920906-160921506	1.44E-21	0	3	Olfactory receptor 81
Olr821	NM_001000842	chr4:70985769-70986369	3.28E-11	1	1	Olfactory receptor 821
Trip13	NM_001011930	chr1:30162343-30163033	4.06E-08	1	0	Thyroid hormone receptor interactor 13
Vom1r29	AY510346	chr1:63196521-63197705	3.75E-18	2	3	vomeronasal 1 receptor, 29
Vom1r3	AY510342	chr1:57221331-57221931	3.17E-13	1	5	vomeronasal 1 receptor 3
Vom1r56	AY510280	chr1:73406816-73407416	3.38E-10	1	1	vomeronasal 1 receptor, 56
Vom1r84	AY510312	chr4:86887717-86888419	2.05E-13	0	2	vomeronasal 1 receptor, 84
Vom1r86	AY510308	chr4:86950403-86951088	3.40E-11	1	2	vomeronasal 1 receptor, 86
Vom1r87	AY510311	chr4:87006096-87006786	1.93E-08	1	1	Vomeronasal 1 receptor 87
Vom1r93	U36896	chr4:123911382-123911982	3.12E-09	3	1	vomeronasal 1 receptor, 93
Vom2r11	NM_001099470	chr1:57382648-57383341	2.68E-08	2	2	Vomeronasal 2 receptor 11
Vom2r36	NM_001099483	chr1:73188300-73188900	4.39E-11	2	1	Vomeronasal 2 receptor 36
Vom2r60	NM_001099480	chr12:1140789-1141389	7.98E-08	2	2	Vomeronasal 2 receptor 60
**Signaling**						
Brsk1	NM_001127337	chr1:67879661-67880261	9.96E-19	1	0	BR serine/threonine kinase 1
Defa9	AY623753	chr16:75272675-75273275	6.03E-10	1	0	Defensin alpha 9
Eepd1	NM_001014088	chr8:25170198-25170876	3.27E-14	1	3	Endo-/exonuclease/phosphatase family domain 1
Frmpd4	NM_001106960	chrX:47679803-47680403	2.02E-10	2	0	FERM and PDZ domain containing 4
Gpaa1	NM_001004240	chr7:114375307-114376102	1.22E-09	1	4	Glycosylphosphatidylinositol anchor attachment 1
Hrsp12	NM_031714	chr7:69940911-69941511	2.41E-23	2	0	Heat-responsive protein 12
Iqcg	NM_001014230	chr11:69550989-69551670	3.81E-08	0	2	IQ motif containing G
Plekhg2	BC169013	chr1:83483036-83483636	1.96E-08	4	0	Pleckstrin family G member 2
Pik3c3	NM_022958	chr18:22494497-22495193	2.29E-13	3	3	Phosphoinositide-3-kinase, class 3
Sel1l2	NM_001014049	chr3:128562470-128563250	1.06E-10	0	1	Sel-1 suppressor of lin-12-like 2
**Transcription**						
Ccdc7	NM_001011561	chr19_r:835125-835824	1.66E-20	4	0	coiled-coil domain containing 7
Ciz1	NM_001106568	chr3:11486246-11486846	6.90E-43	2	2	CDKN1A interacting zinc finger protein 1
Dnajc30	NM_001109024	chr12:22726326-22726926	4.69E-10	3	1	DnaJ (Hsp40) homolog, subfamily C, member 30
Fbxo15	NM_001108436	chr18:81502980-81503580	5.97E-11	2	1	F-box protein 15
Mina	NM_153309	chr11:41651274-41651977	5.97E-08	1	0	Myc induced nuclear antigen
Nfyb	NM_031553	chr7:23187814-23188499	1.38E-57	3	0	Nuclear transcription factor-Y beta
Nop16	NM_001047095	chr17:16084544-16085239	1.38E-13	1	0	NOP16 nucleolar protein homolog
Polr3g	NM_001109468	chr2:9807293-9807893	5.07E-30	2	6	Polymerase (RNA) III polypeptide G
Reck	NM_001107954	chr5:60339205-60339805	6.79E-08	1	0	Reversion-inducing-cysteine-rich kazal motifs
Snapc4	NM_001108574	chr3:4555001-4555601	1.08E-10	1	0	Small nuclear RNA activating complex, 4
**Translation & Protein Modification**						
Arfip2	NM_001004222	chr1:163372155-163373134	3.76E-13	3	1	ADP-ribosylation factor interacting protein 2
Rpl31	NM_022506	chr9:38437621-38438221	1.97E-08	2	0	Ribosomal protein L31
Rpl35a	NM_021264	chr11:69550989-69551670	3.81E-08	0	2	Ribosomal protein L35a-like
Rtf1	NM_001108958	chr3:106189897-106191081	7.77E-09	1	1	Paf1/RNA polymerase II complex component
Taf9b	NM_133615	chrX:94353049-94354039	1.88E-10	0	1	TATA box binding protein (TBP) 9b
**Miscellaneous & Unknown**						
Brd9	NM_001107453	chr1:30162343-30163033	4.06E-08	1	0	Bromodomain containing 9
RGD1359529	NM_001014193	chr5:153696495-153697095	9.47E-08	3	0	Similar to chromosome 1 open reading frame 63
Sdccag3	NM_001013135	chr3:4555001-4555601	1.08E-10	1	0	Serologically defined colon cancer antigen 3
**ESTs**						
RGD1305537	NM_001108822	chr16:13555220-13555820	4.93E-08	1	1	Similar to RIKEN cDNA 3110001I22

### Overlap between SRY and SOX9 Binding Targets

Considering the 71 direct downstream binding targets of SRY identified and 109 direct binding targets for SOX9, and including questionable positives, only five promoters were common between SRY and SOX9 targets. These common targets are Higd2a, Nop16, Orl770, Rtf1, and Vom2r11. A comparison of atypical binding targets (those without binding motifs) identified only one promoter in common at p<1×10^−7^. Using a less stringent statistical cut off (p<1×10^−5^), we found 175 in SRY ChIP and 259 in SOX9 as direct binding targets. Of these less stringent binding targets, only 13 overlapped. An analysis of the overlap between direct and atypical binding targets was performed. Between direct binding targets of SRY and atypical binding targets of SOX9, there were none at both p<1×10^−5^ and p<1×10^−7^ cut-offs, whereas between SRY atypical and SOX9 direct binding targets there were 15 promoters in common, Table 2. Therefore, some atypical binding targets of SRY may be the direct binding targets for SOX9. Interestingly, combined observations demonstrate minimal (<10%) overlap between SRY and SOX9 targets suggesting the two factors are functionally distinct in the induction and progression of Sertoli cell differentiation and testis development.

### SRY and SOX9 Binding Motifs

**Figure 4:**
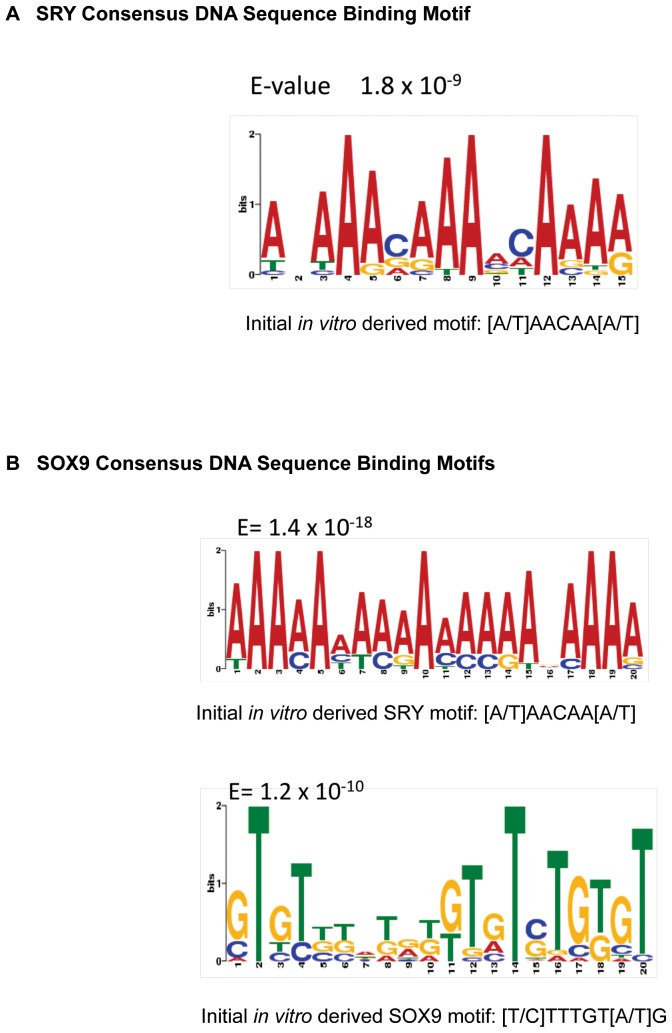
SRY (A) and SOX9 (B) DNA sequence binding motifs. The y-axis indicates the base and size performance for binding, and x-axis the base pair sequence for the motif.

The DNA sequence binding motifs or response elements have been previously described using a limited number of binding sites to investigate the SRY binding motif as [T/A]AACAA[T/C] and [T/A]TTGTT[A/T] and the SOX9 binding motif as [T/C]TTG[T/A]G and [T/A]AACAA[T/A] [22], [31]. Using all the SRY and SOX9 direct binding targets a computerized bioinformatics procedure (Meme Suite) previously described [32] identified the consensus DNA sequence binding motifs. The SRY binding motif is shown in Figure 4A and presents the various nucleotides at each base pair associated with the SRY binding site. The most common nucleotides were similar to the previously described SRY site and gave alternate nucleotides to consider. The SOX9 binding motif is shown in Figure 4B and shows similarity with the most common nucleotides and the previously described SOX9 site [22], [31]. This current analysis has extended previous studies and presents a potentially more accurate consensus SRY and SOX9 binding motifs described due to the use of all major direct downstream binding targets identified. However, further investigation with mutagenesis and in vitro binding analysis is needed to confirm the consensus motifs identified.

### Gene Expression of SRY and SOX9 Binding Target Genes

**Figure 5:**
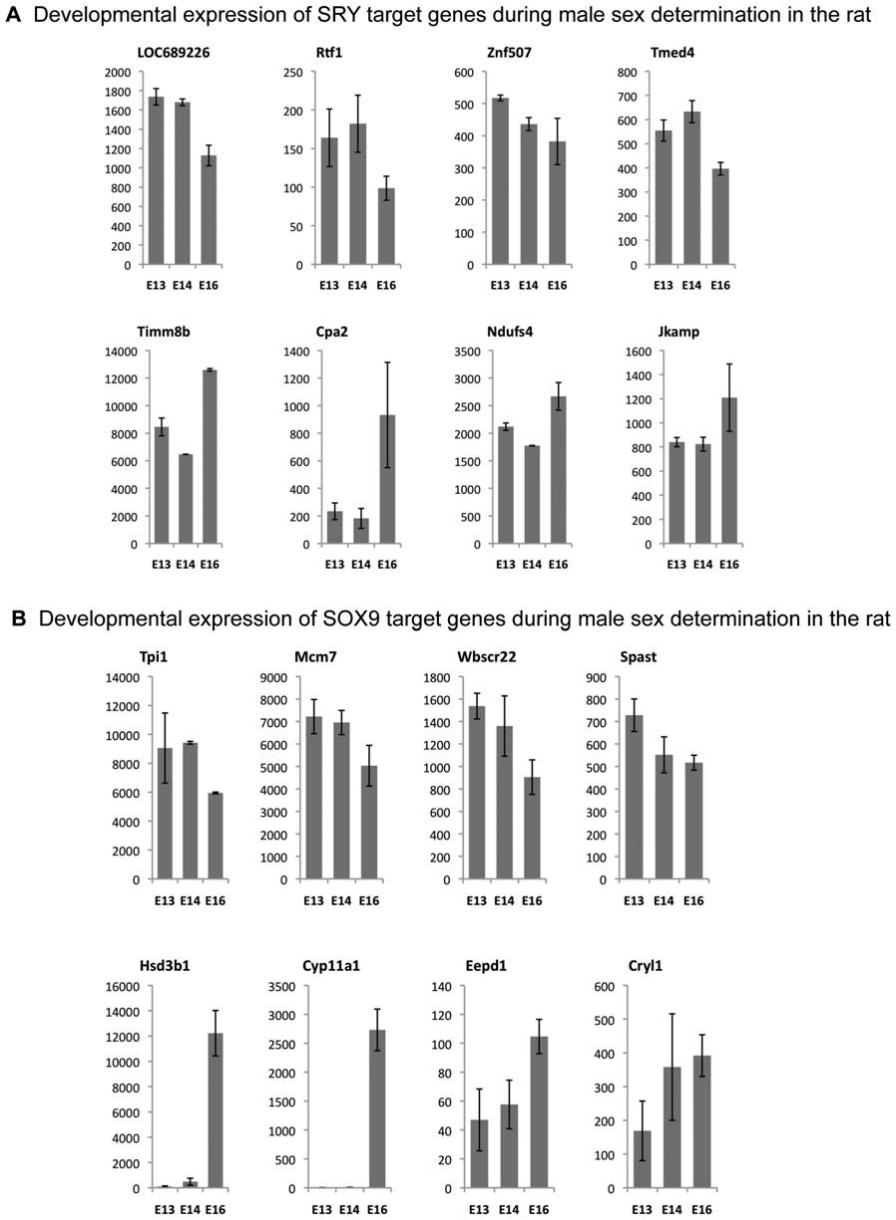
SRY (A) and SOX9 (B) direct binding target gene expression profiles for genes with a statistically (p<0.05) significance change in expression between the developmental periods. Microarray analysis of embryonic day E13, E14 and E16 testis data previously described [26] was used to construct the expression profiles of the selected genes.

Previously the developmental expression pattern of genes in rat and mouse testis at various periods during gonadal sex determination was investigated [33], [34]. Expression patterns of genes that had a statistically significant change in gene expression (p<0.05) from the previous rat microarray analysis were plotted (Figure 5). Expression data indicate that among the genes from the SRY binding target list that eight had a statistically significant (p<0.05) change during male sex determination: Loc689226, Tmed4. Znf507, Rtf3, Cpa2, Jkamp, Timm8b and Ndufs4. Similarly eight from the SOX9 list were changed: Cyp11a1, 3beta HSD1, Tpi1, Mcm7, Wbscr22, Spast, Eepd1, and Cyrl1. Observations do not identify general trends in altered gene express for either SRY or SOX9 response genes. A limitation to this analysis is that the microarray analysis used whole testis, such that Sertoli cell specific expression and regulation could not be assessed.

The current study was designed to identify the binding targets and not assess transcriptional regulation of the binding targets by SRY or SOX9. Since both stimulatory and repressive roles for SRY and SOX9 have been demonstrated, not all binding targets are anticipated to be directly regulated at the developmental time point examined. A preliminary analysis used a previous report involving an E13 gonadal cell culture and a transient transfection of an SRY expression construct to over-express SRY and then assess effects on gene expression with a microarray analysis [27]. From the list of genes with a statistically significant (p<0.05) alteration in gene expression after SRY over-expression 13 were found to overlap with the SRY binding targets identified. The direct binding targets that were induced were Ragl, Rpl24, Fam12b, and Phox2a. The direct binding targets that we suppressed were Exoc4 and RGD1303127. The atypical binding targets that were induced were Thap1, Cd7 and RGD1306839. The atypical binding targets that were suppressed were Chrna3, Id2, F2rl2 and Rspo3. Observations suggest approximately 10% of the SRY direct binding targets are transiently regulated in the E13 gonadal cell culture by SRY. Future studies will need to be directed at the transcriptional regulation of the downstream targets identified.

### Functional Gene Categories, Pathways and Network Analysis

**Figure 6:**
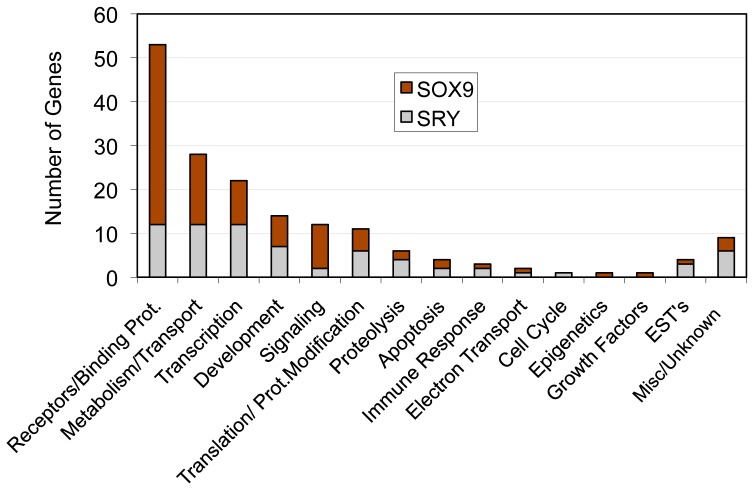
Functional gene categories for SRY and SOX9 direct binding target genes, number of representative genes per functional category listed are indicated.

Analysis of specific functional gene categories combined both the SRY and SOX9 direct downstream binding target genes. As shown in Tables 1 and 2, all the genes were presented in specific functional categories. The general functional categories for both the SRY and SOX9 gene sets are summarized in Figure 6. The cellular receptors and binding proteins, metabolism and transport and transcription were the most abundant categories for both SRY and SOX9 targets. In order to understand whether SRY and SOX9 direct binding targets are enriched for specific cellular signaling pathways the SRY and SOX9 target genes were analyzed with a KEGG pathway analysis as described in the Methods. The analysis identified significant enrichment for several signaling pathways including: olfactory transduction, RNA processing, metabolic pathways, steroid hormone biosynthesis, protein processing in endoplasmic reticulum, oxidative phosphorylation, and several disease pathways (Table 3). Olfactory transduction was the most highly enriched pathway for both SRY and SOX9 targets. This was due to the large number of olfactory receptors identified, Supplemental Figure S3.

**Table 3:** Cellular Pathways Enrichment for Sox9 and Sry.

	Number of Affected Genes	
Pathway Name	Sox9	Sry
Olfactory transduction	16	7
Ribosome	5	1
Tryptophan metabolism	4	
Inositol phosphate metabolism	3	1
HTLV-I infection	3	
Neurotrophin signaling pathway	3	
Steroid hormone biosynthesis	3	
Protein processing in endoplasmic	2	3
Huntington's disease	2	3
Alzheimer's disease	2	3
Oxidative phosphorylation	1	3
Parkinson's disease	1	3

**Figure 7:**
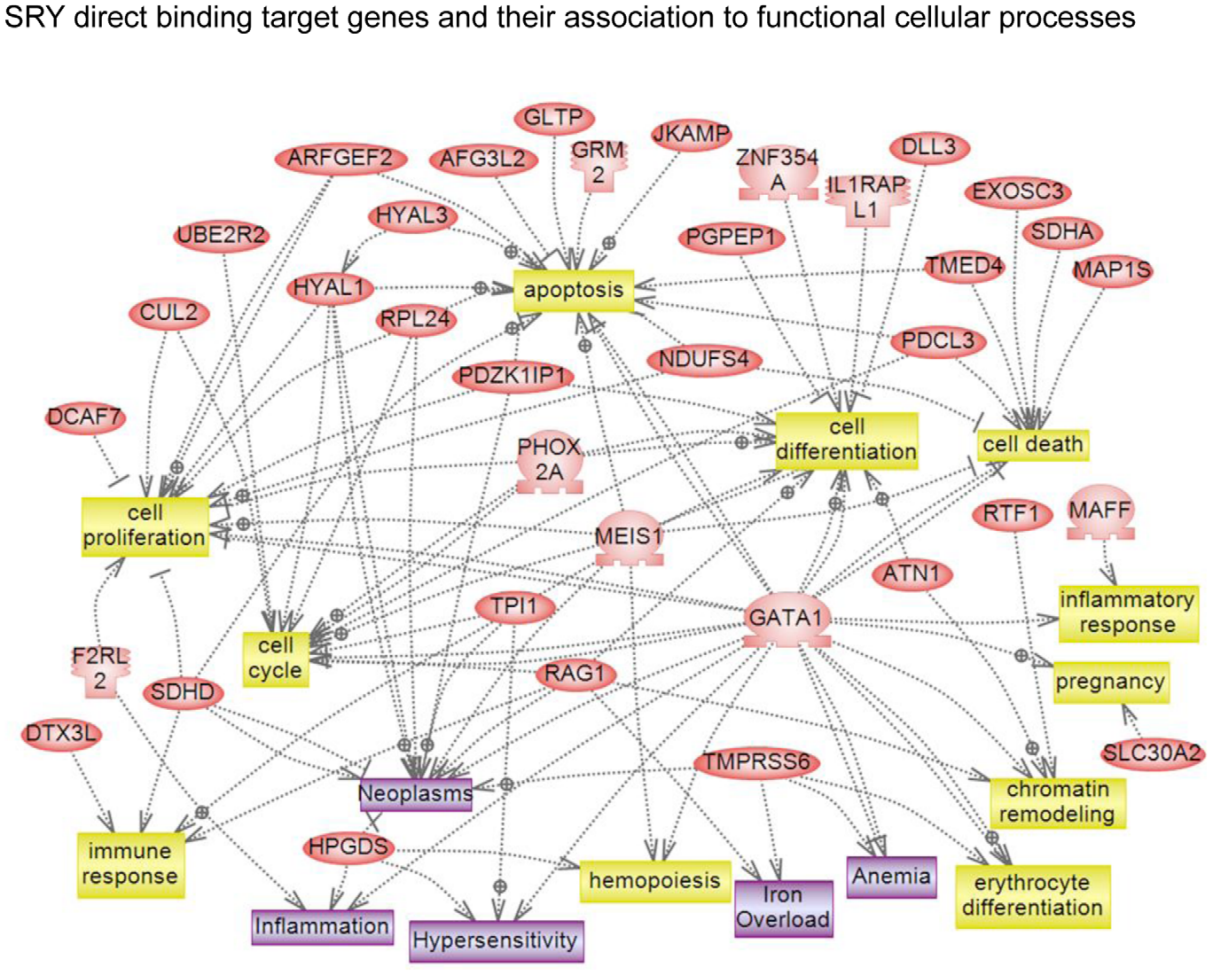
Gene network of shortest connections to cellular processes for 71 direct downstream gene targets of SRY, as obtained by global literature analysis using Pathway Studio 7.0 (Ariadne Genomics, Inc., Rockville, MD).

**Figure 8:**
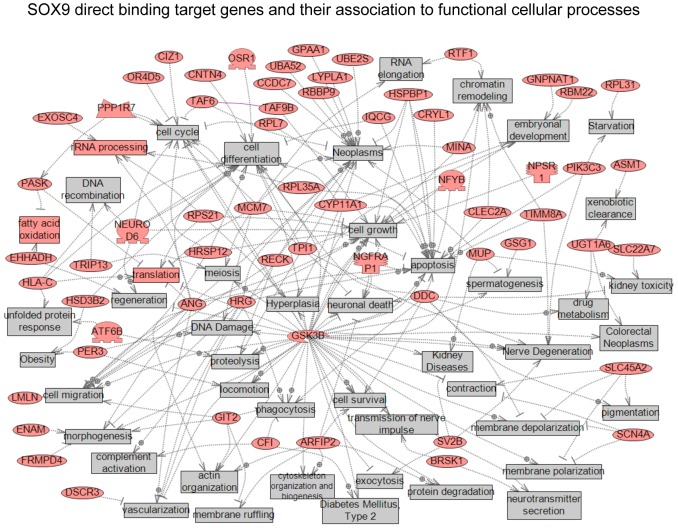
Gene network of shortest connections to cellular processes for 109 direct downstream gene targets of SOX9, as obtained by global literature analysis using Pathway Studio 7.0 (Ariadne Genomics, Inc., Rockville, MD).

A gene network analysis of SRY and SOX9 direct binding target genes identified specific gene networks. No significant direct interaction (connection) gene networks were identified for either SRY or SOX9. In contrast, indirect connection gene networks with associations with various cellular processes were identified. The SRY target genes that have indirect associations showed connections with cell proliferation, apoptosis, cell differentiation, cell death, cell cycle, chromatin remodeling and immune response, Figure 7. SOX9 binding target genes had more complex associations with cell differentiation, cell proliferation, embryonic development, cell cycle, DNA processing, spermatogenesis, cell growth, chromatin remodeling, cell survival, cell migration, and DNA damage, Figure 8. Although indirect connection gene networks were identified, no specific pathways or cellular processes were predominant. The SOX9 direct binding targets had a much larger number of associated cellular processes than the SRY binding targets.

### Comparison of Literature-based SRY Target Genes

Although SRY has been shown to have multiple functions, only a handful of downstream targets have been previously identified. Comparison of the complete SRY and SOX9 direct target lists with the published literature identified five genes from Bradford et al. 2009 [25] that were found to overlap with the complete target gene list. Among those overlapped, Adam23 was the one that is a direct target of SRY and the other four (*Cbln4*, *Nr5a1*, *Flrt2*, and *Tmem95*) were atypical binding targets. The genes that are known to participate in Sertoli cell differentiation such as *Fgf9*, *Gata4*, *Amh* were not found using the statistical p<1×10^−7^ cut-off. These genes are part of the testis differentiation gene network, but may not be directly regulated by SRY. Interestingly, hematopoetic prostaglandin D synthase (*Pdgs*) gene was found to be the direct binding target of SRY at the significant level of p<1×10^−9^. Prostaglandin has been previously found to be regulated by SOX9 [17] and to promote Sertoli cell differentiation.

## Discussion

Jost's sex determination theory [1] states that the sex of an individual will be determined and expressed as 1) chromosomal sex (genetic) in presence or absence of Y chromosome, 2) gonadal sex which is controlled by presence or absence of testis determining factor (TDF), and 3) phenotypic sex which is determined by the hormonal products produced by the gonads. In 1970s and 1980s, several potential TDF in male sex determination were considered including H-Y antigen and ZFY, which was suggested based on mutations in the Y chromosome that led to sex reversal in humans and mice [4], [5], [6], [7]. In 1990, SRY was discovered as the TDF that initiates mammalian testis differentiation from a bipotential gonad [8], [9], [10]. Studies then identified the SRY downstream target SOX9 as facilitating Sertoli cell differentiation and male gonadal sex determination [11], [12]. The *in vivo* downstream targets of these two master regulators of male sex determination have eluded scientists for decades. The current study was designed to identify the *in vivo* downstream binding targets of both SRY and SOX9 using a genome-wide ChIP-Chip comparative hybridization procedure. These two key factors were found to interact with distinct developmental gene networks during testis differentiation.

A chromatin immunoprecipitation (ChIP) with antibodies specific for SRY or SOX9 was performed as previously described [27]. Due to the potential of non-specific binding of IgG to chromatin, we performed a comparative hybridization of SRY or SOX9 antibody ChIP DNA with a non-immune IgG ChIP DNA to eliminate the potential of detecting false positive SRY or SOX9 targets. As discussed, the negative ChIP-Chip peaks identified demonstrate a relatively high degree of non-immune IgG binding that needs to be considered in assessing ChIP-Chip data. The questionable binding targets identified were those that had a positive peak in the ChIP-Chip adjacent to a negative IgG peak. Therefore, the current procedure of a ChIP-Chip comparative hybridization with a non-immune IgG control reduces the false positive in the analysis. However, the potential that an IgG non-specific peak may mask a true positive ChIP-Chip peak is a limitation of this approach. The downstream candidates identified are likely a subset of a number of downstream binding targets that remain to be identified. Therefore, some anticipated targets may not be present due to this technical limitation.

SRY has been proposed to have several functions including the repression of testis repressors [35], chromatin remodeling [36], pre-RNA splicing [37], and transcriptional activation [38], suggesting that there might be a number of different target genes for SRY. The first functional *in vivo* target gene that was identified and studied is SOX9. SOX9 does regulate several downstream pathways underlying differentiation of embryonic testicular somatic cells [22] and inhibition of meiosis in the germ cells [39], [40], [41]. Interestingly, the current study did not detect SRY ChIP hybridization signals for SOX9 because only approximately 5000 base pairs of the promoter regions were present on the promoter tiling array. The testis specific enhancer of *Sox9* for SRY binding is located at 7 Kb upstream of the transcription start site so could not be detected. Therefore, the presence of *Sox9* enrichment in the SRY ChIP was confirmed and validated by *Sox9* TESCO PCR, Figure 2. The TESCO sequence in the mouse and rat *Sox9* promoters is highly conserved [24] and the PCR data confirmed *Sox9* is a direct downstream target of SRY. This observation with *Sox9* suggests SRY targets outside the 5 Kb promoter region will not be detected, such that the current SRY downstream target list is a subset of a potentially larger list that remains to be determined. An additional validation of the SRY ChIP-Chip analysis used two recently identified direct targets for SRY. The ChIP-Chip arrays identified the presence of *Tcf21* in the SRY ChIP [27], Figure 1. Our previous studies have also found *Ntf3* a direct downstream target of SRY in the rat [26]. Both *Tcf21* and *Ntf3* have distinct functions in testicular morphogenesis [28], [29], [42] and were detected in the SRY ChIP-Chip assay. Therefore, the detection of these known SRY targets in the ChIP-Chip assay helps validate the protocol used.

Another previously identified SRY downstream target gene is Cbln4 [25] and was found to be one of the downstream targets of SRY, but not of SOX9. The sequences of the Cbln4 induced by the SRY-enriched ChIP DNA fragment did not have a putative SRY-binding motif, so we categorized Cbln4 as an atypical binding target of SRY. Previously Cbln4 was reported to be one of the downstream targets of SRY based on cloning of the *in vivo* ChIP DNA [25]. In the current study the ChIP-Chip assay required at least three biological replicates of the ChIP experiments to be positive while the IgG background signals of hybridization are bioinformatically removed. The stringency of our ChIP protocol (absence of protein cross-linking) and bioinformatics analysis will likely eliminate some SRY targets, but we are confident that the direct binding target lists reported are accurate.

Following the analysis of the SRY ChIP data, promoter regulatory regions that were enriched with the *in vitro*-derived DNA binding motif [A/T]AACAA[A/T] were termed as direct downstream binding targets. Similar analysis for the SOX9-associated regions identified the same binding motif as shown above and in addition a consensus of Sox9-specific *in vitro*-derived binding motif [17]. Since a large number of downstream direct binding targets were identified for SRY and SOX9, we performed a sequence motif analysis of all the sites and developed a refined DNA sequence binding motif for both SRY and SOX9. These initial motifs overlapped well with the new motifs, but the new motifs have expanded the variability and significance of specific nucleotides. These new SRY and SOX9 binding motifs were utilized by all the direct downstream binding targets identified. The regions that overlapped between SRY and SOX9 target sequences contained predominantly SRY-specific HMG motif indicating that SOX9 interacts with DNA with a higher degree of variability and differently from SRY binding to its consensus motif. Future analysis of the SRY and SOX9 consensus motif identified will require mutagenesis and in vivo binding experiments to confirm the functional role of these motifs. The majority of the ChIP enriched regions of the SRY binding targets did not contain an SRY consensus motif, suggesting that SRY can act indirectly in transcriptional complexes not involving direct binding to the DNA target. In contrast, 80% of the SOX9 targets contained a consensus binding motif [9], suggesting a more direct binding role for SOX9 in regulation of downstream targets. The indirect actions of SRY on the atypical binding targets potentially expand its functional role in regulating genome activity and testis development.

Interestingly, observations demonstrate SRY and SOX9 have different downstream binding targets with minimal (<10%) overlap. Previous suggestions were made that SOX9 would singularly replace SRY during development, but the current observations suggest *Sry* and *Sox9* have distinct roles during development. Therefore, SRY induces a cascade of transcriptional events that do not simply have SOX9 to replicate SRY actions. However, the possibility that SRY and SOX9 may act at distinct targets, but influence similar cellular pathways and gene networks was considered. Similar functional categories of genes were observed in both the SRY and SOX9 direct binding targets, Figure 6. Therefore, more detailed analysis of specific signaling pathways and gene networks was performed. The only predominant signaling pathway represented by both SRY and SOX9 was the olfactory transduction pathway, Table 3. However, all the genes in this pathway were olfactory receptors, Supplemental Figure S3, and the functional impact of such a pathway is difficult to assess during testis development. No other major specific pathways were in common between SRY and SOX9. A final consideration used a gene network analysis to determine if direct connection (interaction) genes were common between SRY and SOX9. No significant direct connection gene networks were affected by SRY or SOX9. Therefore, the genes in the downstream direct binding targets did not have significant functional links or connections. Subsequent gene network analysis with SRY and SOX9 direct downstream binding target genes for indirect associations with functional cellular processes revealed SRY and SOX9 did have common connections to several processes, such as cell differentiation, cell proliferation, chromatin remodeling, cell death, and apoptosis. In contrast to the limited number of direct connections (interactions) within SRY or SOX9 target genes, a large number of associations were observed with cellular processes. Similar indirect associations have been reported for DMRT1 regulated genes in postnatal testis where DMRT1 recognizes a SOX9 motif like element for binding [43]. Combined observations identify the primary cellular processes and functional categories affected by SRY, but limited overlap was observed with SOX9 binding targets. Therefore, the downstream targets of SRY suggest a large number of different genes effect a wide variety of processes with no underlying gene network involved.

Analysis of the gene expression of the SRY and SOX9 direct downstream targets used our previously published rat testis development microarray database at embryonic day E3, E14 and E16 [33], [34]. The majority of SRY and SOX9 direct target genes did not have altered gene expression during the fetal developmental period investigated. A limitation of these gene expression observations is that whole embryonic gonads were used in the analysis which contain multiple cell types. Therefore, Sertoli cell gene expression was not assessed directly and the expression data is due to a combination of cells. This could mask gene expression changes or suggest an alteration when changes in cell populations develop (e.g. Leydig). Observations suggest there are no major gene expression changes of the SRY or SOX9 target genes during this developmental period, but future studies will need to examine purified populations of Sertoli cells and downstream expression cascade affects. Expression results suggest that SRY target genes *Cpa2* and *Timm2b* were suppressed during early periods, but increased dramatically later. In contrast, SRY targets *Loc869226*, *Tmed4*, *Znf507*, *Rtf1* and *Id2* showed decreasing patterns during the three day window of rat testicular differentiation. A number of genes (*3beta-HSD, Cyp11a1, Insl3*) that could contribute to prepubertal Sertoli cell steroidogenesis were found as direct downstream targets of SOX9.

Although the current study was designed to identify the binding targets for SRY and SOX9, no information is provided on the transcriptional regulation of the binding targets identified. Future studies involving the transcriptional regulatory actions of SRY and SOX9 on these targets are needed. As a preliminary experiment a previous report that used E13 rat gonadal cell culture and transient transfection of an SRY-expression construct to over-express SRY was used to examine regulated gene expression with a microarray analysis [27]. Comparing the regulated gene set from this analysis with the current SRY binding targets demonstrated 13 genes appear to be directly regulated by SRY. Since SRY and SOX9 are known to also have a gene repression role, chromatin remodeling role, and RNA processing role, not all the SRY or SOX9 binding targets are anticipated to be regulated in a SRY over-expression study. However, this preliminary analysis supports the transcriptional regulatory role of SRY on a number of the binding targets identified.

The tilling array used for this experiment represents only 5 kb of the proximal regions of the promoter sequences which represent less than 5% of the rat genome. In the current study, SRY-bound regions from the non-promoter areas were not assessed. In the future it is important to perform whole genome ChIP-Chip or ChIP-Seq (i.e. next generation sequencing) experiments to determine how many additional regions in the genome are bound by SRY and SOX9. These regions might represent tissue specific enhancers for the target gene expression, similar to the TESCO sequence located 7 kb upstream of the Sox9 promoter and involved in regulation of *Sox9* expression by SRY [24]. Therefore, the SRY and SOX9 downstream direct targets identified represent a subpopulation of a potential larger gene set that remains to be elucidated.

The combined observations identify genome wide *in vivo* targets of SRY and SOX9 as an initial attempt to define the mechanisms of male sex determination at the systems level. A large number of direct downstream binding targets were identified for both SRY and SOX9, which were distinct with minimal overlap. Therefore, SRY and SOX9 have distinct functions during testis development involving different downstream targets. The downstream targets influenced a variety of cellular pathways and processes, but did not involve unique gene networks, so acted independently to influence more global processes involving induction of Sertoli cell differentiation. Refined DNA sequence binding motifs were identified for SRY and SOX9 that help explain the distinct actions of SRY versus SOX9. Observations provide insights into the molecular control of testis determination initiated by SRY and induction of Sertoli cell differentiation. Current findings widen the arena for further systems biology investigations of the role of the SRY.

## Materials and Methods

### Tissue preparation

Harlan Sprague-Dawley rats were used for the study. All the rats were kept in a temperature controlled environment and given food and water *ad libitum*. Estrous cycles of female rats were monitored by cellular morphology from vaginal smears. Rats in early estrus were paired with males overnight and mating confirmed by sperm positive smears, denoted day 0 of pregnancy. Pregnant rats were euthanized at embryonic day 13 (E13) of pregnancy, and embryonic gonads were collected for chromatin immunoprecipitation. Sex was determined by PCR using primers specific for *Sry* on genomic DNA isolated from embryo tails as previously described [44]. All procedures were approved by the Washington State University Animal Care and Use Committee (IACUC approval # 02568-026).

### In vivo Chromatin Immunoprecipitation (ChIP) Assay

A modified ChIP (cChIP) assay was adopted from O'Neill et al., (2006) [45] and performed according to Bhandari et al (2011) [27]. The conditions for the native-ChIP (not including cross linking) were optimized for immunoprecipitating with SRY and SOX9 antibodies. The native-ChIP was used to identify high affinity binding sites and reduce low affinity sites, and was validated with PCR for *Sox9, Tcf21* and *Ntf3*. To run a replicate of the ChIP assay, at least twenty male gonads from thirty 13 dpc (12–18 tail somite stage) rat embryos were used per array. All three ChIP experiments were with different biological samples. *Drosophila* SL2 cells (American Type Culture Collection (ATCC) Catalog no. CRL-1963) were used as a carrier. Densely-grown cells (approximately 5×10^7^ cells) were pelleted and washed three times in ice-cold phosphate buffered salve (PBS), 5 mM sodium butyrate and resuspended in 0.5 ml NB buffer (15 mM Tris-HCL, pH 7.4, 60 mM KCl, 15 mM NaCl, 5 mM MgCl_2_, 0.1 mM EGTA, 0.5 mM 2-mercaptoethanol, 0.1 mM PMSF). Testis samples were mixed with SL2 cells and homogenized to make single cell suspension. Nuclei were pelleted, resuspended in 10 ml NB buffer, 5% (vol/vol) sucrose, pelleted and resuspended again in 1.5 ml digestion buffer (50 mM Tris-HCl pH 7.4, 0.32 M sucrose, 4 mM MgCl_2_, 1 mM CaCl_2_, 0.1 mM PMSF). Following micrococcal nuclease digestion (NEB, USA) for 5 minutes at 28°C, the digested samples were gently spun (800× g) for 15 minutes and supernatant set aside on ice. The pellet was resuspended in 250 ul digestion buffer and again centrifuged gently at 800× g for 15 minutes at 4 degrees. Both the supernatants were pooled and a fraction (50 ul) out of it was kept aside to use as input. The remaining supernatant was incubated with either non-immune IgG or anti-SRY (Santa Cruz, CA, USA) or anti-SOX9 (ABcam, CA, USA) antibodies at 4°C overnight. The specificity of these antibodies on western blots have been previously described and validated [27].

After incubation with 100 µl of pre-swollen protein A-Sepharose beads (SL2 DNA blocked) for 2 h at 4°C, the bead-bound immunoprecipitates were centrifuged gently and washed five times with wash buffer (50 mM TrisHCl pH 7.5, 10 mM EDTA, 5 mM Na butyrate and 50–150 mM NaCl). The protein-DNA complexes were incubated at room temperature with elution buffer (1% SDS in TE) and centrifuged at 11500× g for 10 minutes. Elution was repeated two times and eluted DNA was pooled. Co-immunoprecipitated DNA was purified by phenol/chloroform extraction, and ethanol precipitation. Final concentration of immunoprecipitated DNA varied from 200 to 500 ng per assay. Three different experiments and ChIP assays were performed. Exactly 30 ng of immunoprecipitated DNA from each assay was amplified by whole genome amplification kit developed by Sigma (Sigma #WGA2 50 RXN). At least five separate whole genome amplifications were performed and DNA was pooled. Pooled whole genome amplified DNA was purified by using Promega's Wizard SV40 PCR cleanup kit (Promega). Purified DNA was checked on the gel and sent to Nimblegen for ChIP-chip hybridization (Nimblegen, Iceland). A three plex array (3×720 RefSeq Promoter Array) was used for hybridization comparisons. Confirmation of ChIP immunoprecipitation was done in a PCR by using primers against Tcf21 promoter [27] and SOX9 TESCO as previously described [26], [27]. Confirmation of the selected candidate genes were done by semi-quantitative PCR method with primers listed in Supplemental Table S4.

### Bioinformatics Analysis of ChIP-Chip Data

The ChIP-Chip hybridization used a Roche Nimblegen's Rat ChIP-chip 3×720K RefSeq Promoter Array. The enrichment for each probe on the array was calculated as the log ratio of the intensities of hybridization for SRY or SOX9 ChIP DNA (Cy5) to control DNA from IgG control (Cy3). Array contained on average 4,000 bp of promoter for each of 15,287 promoters in the rat genome corresponding to 15,600 RefSeq transcripts (approximately 3880 bp upstream and 970 bp downstream from transcription start site). The analysis of ChIP-chip data was performed as previously described [32]. For each hybridization experiment raw data from both the Cy3 can Cy5 channels were imported into R (R Development Core Team (2010), R: A language for statistical computing, R Foundation for Statistical Computing, Vienna, Austria. ISBN 3-900051-07-0, URL http://www.R-project.org), and data checked for quality and converted to MA values (M = Cy5-Cy3; A = (Cy5+Cy3)/2). The R codes that were used for analysis and annotation are available in the following website: http//www.skinner.wsu.edu. All the tiling array Chip data was deposited in the NCBI GEO site (GEO# pending).

Within each array, probes were separated into groups by GC content and each group was separately normalized using the LOESS normalization procedure [46]. This allowed for groups with optimal GC content, which exhibited a reduced quality issue, to receive a normalization curve specific to that group. After each array was normalized within array, the arrays were then normalized across arrays using the A-quantile normalization procedure [47]. Following normalization the probe's normalized M values (and then A) were replaced with the median value of all probe M values (and then A) within a sliding window of 600 bp [48], [49], [50], due to the size of DNA fragments used. Following normalization each probe's M value represents the median intensity difference between Cy5 and Cy3 of a 600 bp window. Significance was assigned to probe differences between experimental (SRY or SOX9) and IgG control by calculating the median value of the intensity differences as compared to a normal distribution scaled to the experimental mean and standard deviation of M. Regions of interest were then determined by combining consecutive probes with significance p-values less than 10^−3^. Significance was assigned to probe differences between experimental and control by calculating the median value of the increasing differences as compared to a normal distribution scaled to the experimental mean and standard deviation of the mean. A Z-score and P-value were computed from that distribution with the use of R code analysis. The statistically significant peaks of hybridization were identified and P-value associated with each peak presented. Each peak of interest was then annotated for the gene. Every promoter exceeding the intensity threshold was considered positive for SRY or SOX9 binding. The final list of SRY or SOX9 targets includes the promoter-proximal regions that made the threshold in an average of the three replicates. Hybridization signals for all the candidate promoters that were within the cutoff line (p≤1×10^−7^) were plotted (average of the three replicates), Supplemental Figure S1. The genes that were not in the list but seemed to be masked by IgG negative signals were designated as questionable positives. These questionable positive promoters were manually chosen and confirmed by PCR, Supplemental Figure S2.

### Gene Network and Pathway Analysis

Gene network analysis identified groups functionally interconnected genes whose expression is linked to cellular processes. In the current study gene networks for both SRY and SOX9 downstream binding target genes were constructed separately using previously published criteria for developmental network analysis [51]. Global literature analysis of various gene lists was performed with Pathway Studio software using BiblioSphere Pathway Edition (Genomatix Software GmbH, Munchen, Federal Republic of Germany) which performs direct gene interaction (connection) analysis and relationship with cellular processes (indirect interactions).

The cellular signaling pathway analysis of direct downstream target genes was performed according to the protocol previously described [51]. The downstream binding targets of SRY and SOX9 and their associations with pathways were analyzed for KEGG (Kyoto Encyclopedia for Genes and Genome, Kyoto University, Japan) pathway enrichment using Pathway-Express, a web-based tool freely available as part of the Onto-Tools (http://vortex.cs.wayne.edu). A program based on literature analysis Pathway Studio (Ariadne, Genomics Inc. Rockville MD) was used to evaluate cellular processes connected to binding targets associated genes. The analysis of statistical over-representation of genes within a pathway used a Fisher's Exact test using 2×2 contingency table. The pathway analysis is distinct from the gene network analysis described above.

### Developmental Expression Profiles and SRY Over-expression Analysis of Select Binding Target Genes

The direct downstream binding targets of SRY and SOX9 were separately compared with lists of genes in our previous microarrays from rat developmental studies [34]. The expression profiles of each gene from the ChIP-chip list of downstream targets that had a significant (p<0.05) change in expression were presented.

The influence of SRY over-expression on the downstream binding target genes was evaluated from a previously published microarray analysis on E13 gonadal cell cultures [27]. The E13 rat gonadal cells were cultured and transiently transfected with an Sry expression plasmid to then examine effects on gene expression six days after transfection with a microarray analysis. The SRY binding targets with a statistically significant (p<0.05) change in gene expression after SRY over-expression were identified and presented.

### PCR Confirmation of Select Binding Target Genes

Primers were designed for at least 10 statistically significant binding targets from the region of probe hybridization peaks and tested using WGA-amplified ChIP DNA in a PCR (ChIP-PCR). Primers are listed in Supplemental Table S5. We used non-immune IgG as a negative control in all our ChIP-PCR assay replicates. Data were analyzed by subtracting negative and background signals. Experiments were designed to contain input as a positive control, IgG as a negative control and ChIP enrichment by specific antibody as an experimental. PCR results with positive bands from at least two biological replicates of the ChIP were considered positive and presented.

## Supporting Information

Figure S1 **SRY downstream direct binding target gene promoters.** The positive hybridization is specific to SRY ChIP-DNA signal and negative hybridization to the non-immune IgG ChIP-DNA signal. Hybridization signals are the average of three biological replicates of ChIP assays. Hybridization signals below the statistical significance of p<1×10^−7^ was not considered. Data represent assays from three different experiments and biological replicates.
(PDF)

Figure S2 **Hybridization signals masked by adjacent larger peaks of IgG binding.** (A) *Hes2* hybridization profile with horizontal bar with arrows identifying SRY binding site and PCR confirmation of SRY with anti SRY (aSRY), IgG and input DNA. (B) *Scn4a* hybridization profile with horizontal bar for SOX9 binding and ChIP-PCR confirmation of *Sox9* (aSOX9). ChIP DNA from IgG represented negative control (IgG) in PCR. PCR was conducted on 200 ng DNA amplified by whole genome amplification kit (Sigma). Data represent ChIP-PCR assay from three different experiments and biological replicates.
(PDF)

Figure S3 **Olfactory transduction signaling pathway from KEGG pathway.** The SRY and SOX9 direct binding targets are listed in the olfactory receptor insert box.
(PDF)

Table S1 **Atypical downstream binding targets of SRY during male sex determination in the rat.** Atypical targets were pulled down by SRY antibody, but the hybridization occurred through indirect binding as the peak of hybridization signal did not contain the SRY consensus motif.
(PDF)

Table S2 **Questionable downstream binding target genes of SRY.** Their hybridization signals were masked by negative binding by IgG, so appeared to be negative in the bioinformatic analysis. These promoters were manually extracted from the database. Confirmation of positive binding in the promoter was done by PCR as shown in the supplemental figure S2.
(PDF)

Table S3 **Atypical downstream binding targets of SOX9 during male sex determination in the rat.** Atypical targets were pulled down by SOX9 antibody, but the hybridization occurred though indirect binding as the peak of hybridization signal did not contain HMG or in vitro derived SOX9 consensus motif.
(PDF)

Table S4 **Questionable downstream targets of SOX9 during male sex determination in the rat.** Their hybridization signals were masked by negative binding by IgG, so appeared to be negative in the bioinformatic analysis. These promoters were manually extracted from the database.
(PDF)

Table S5 **PCR Primers Utilized.**
(PDF)
