# Supplementary material for: Global Genome Analysis of the Downstream Binding Targets of Testis Determining Factor SRY and SOX9
Source: PLoS One. 2012 Sep 12;7(9):e43380. doi: 10.1371/journal.pone.0043380 (PMC3440412; doi:10.1371/journal.pone.0043380)

Supplemental Figure S1

SRY Direct Gene Binding Target ChIP-Chip Hybridization Profiles

Afg312

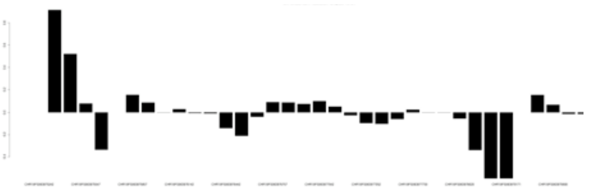

Arfgef2

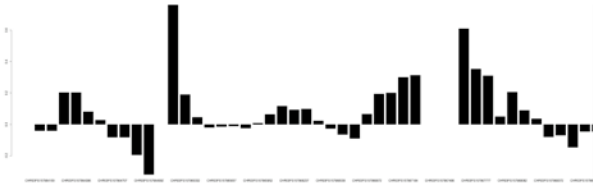

Armc7

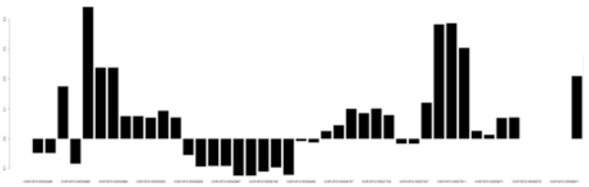

Atn1

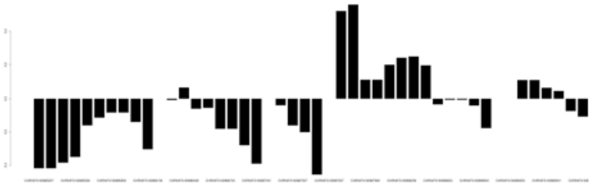

Btbd4

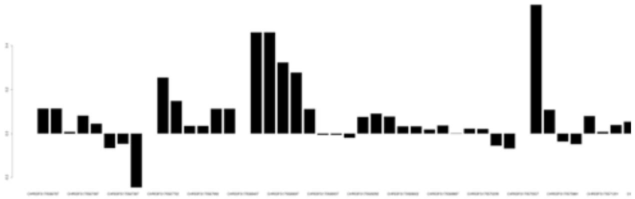

Ccdc127

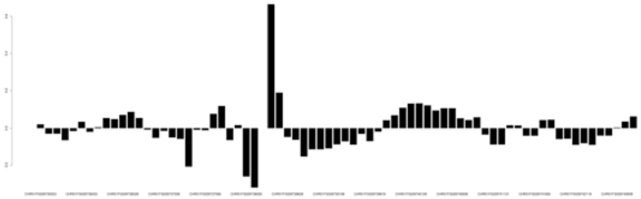

Cd24

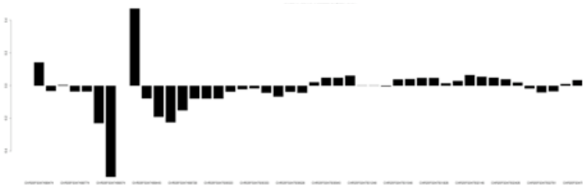

Cpa2

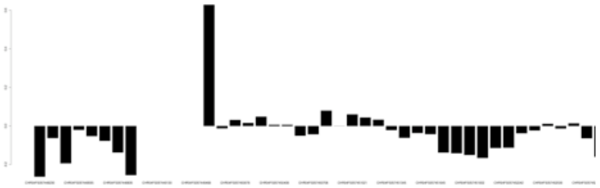

Crygb

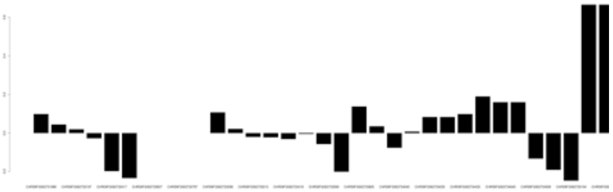

Cul2

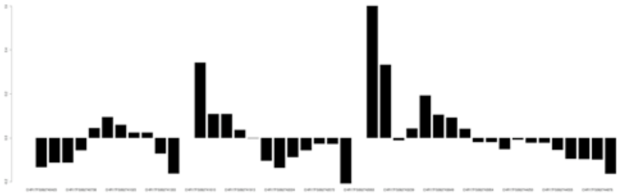

Cytsa

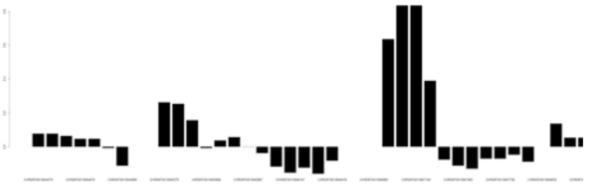

Dcaf7

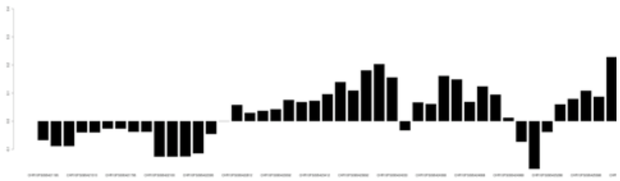

Ddx46

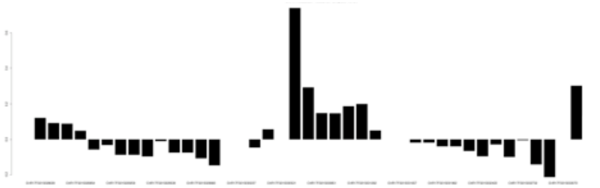

Exoc4

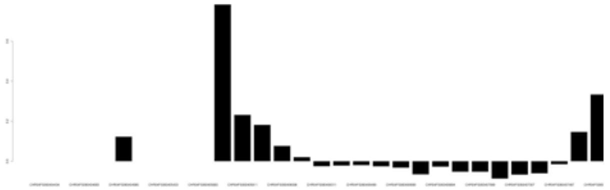

Fam12b

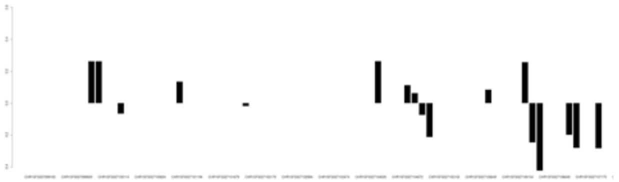

Gata1

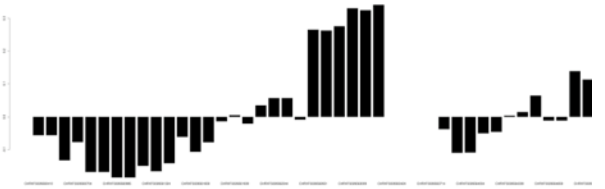

Gltp

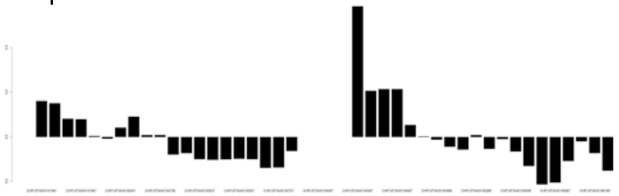

Grm2

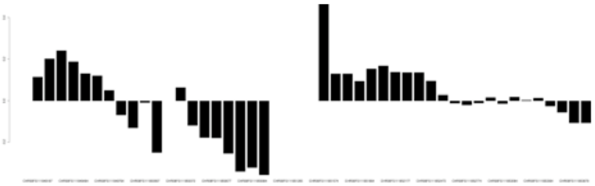

Higd2a

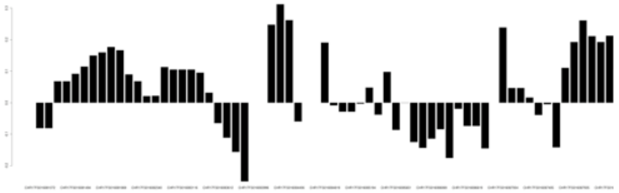

Hpgds

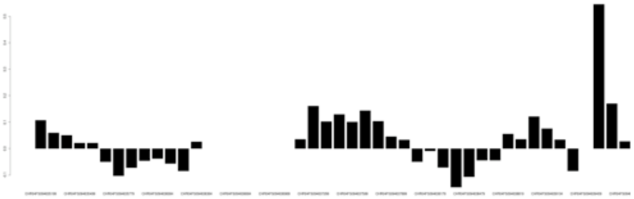

Hyal1

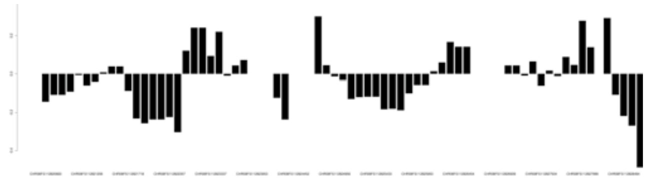

Hyal3

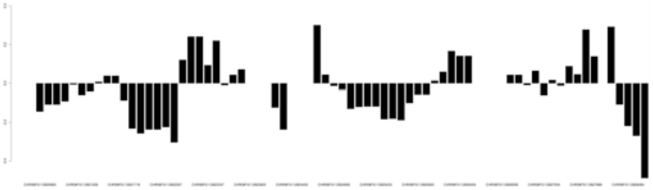

Il1rapl1

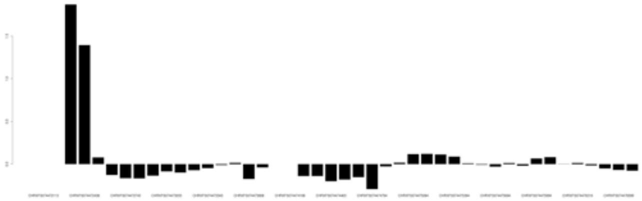

Jkamp

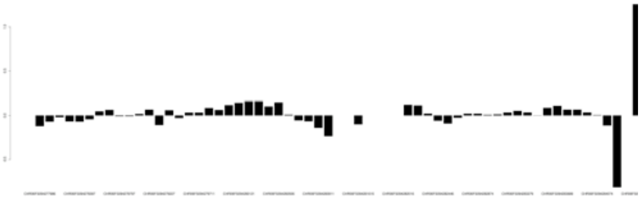

Lcn11

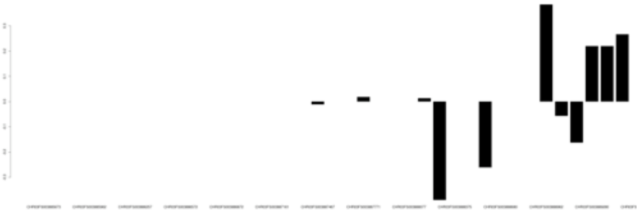

LOC298139

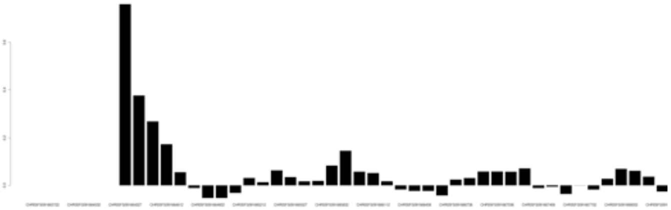

LOC308990

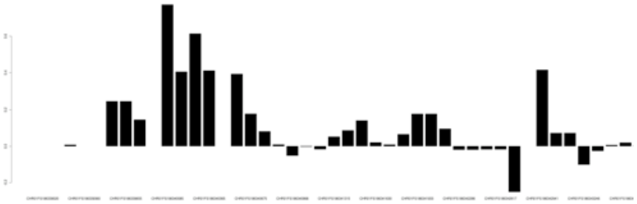

LOC689226

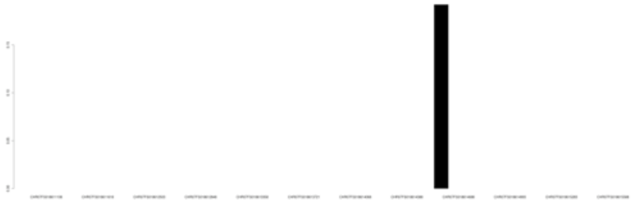

Lrrc68

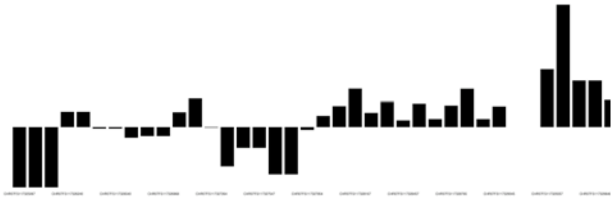

Maff

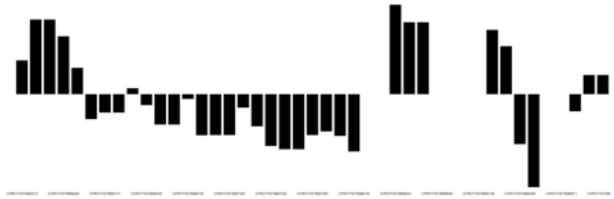

Meis1

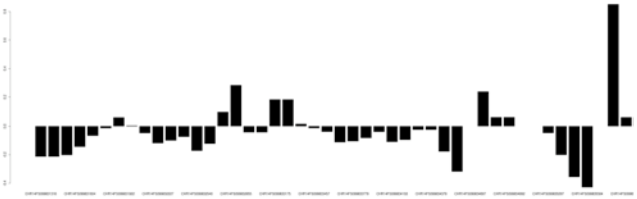

Mrpl51

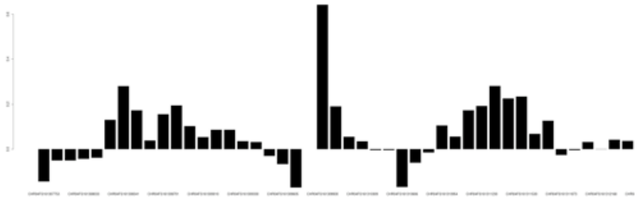

Nop16

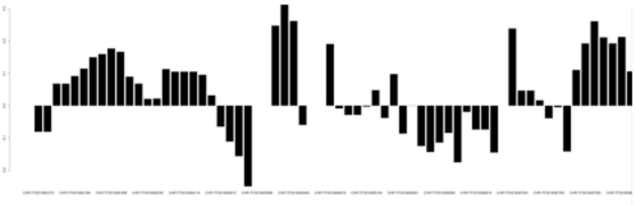

Nsfl1c

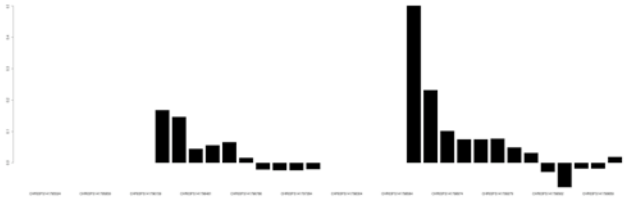

Olr122

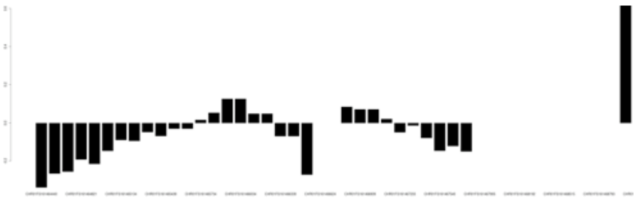

Olr463

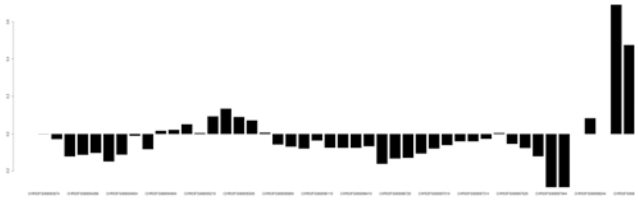

Olr669

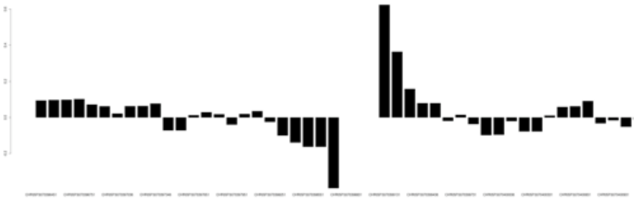

Olr770

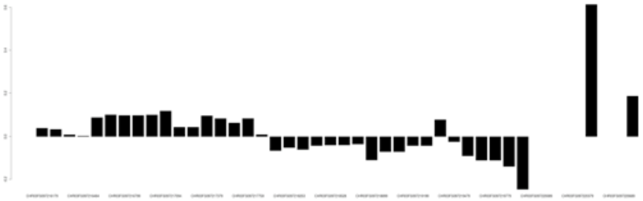

Olr853

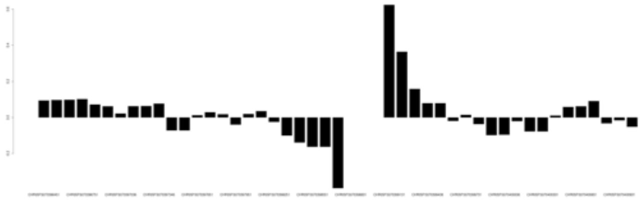

Olr1553

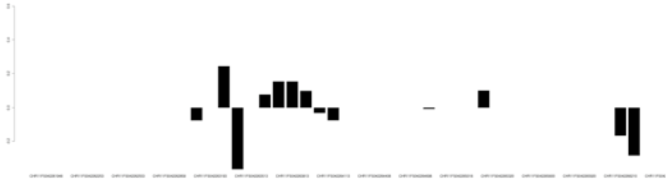

Olr1657

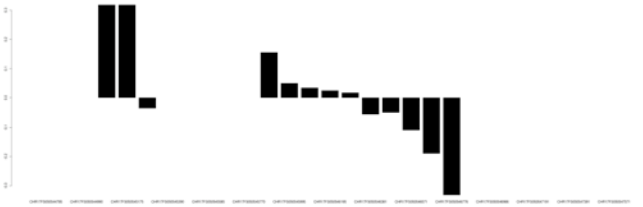

Pdcd6ip

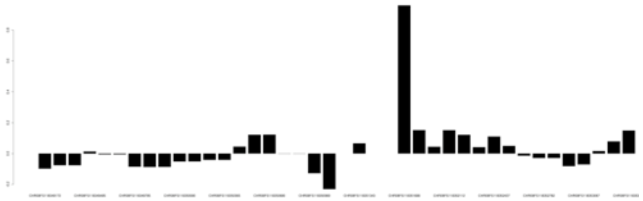

Pdcl3

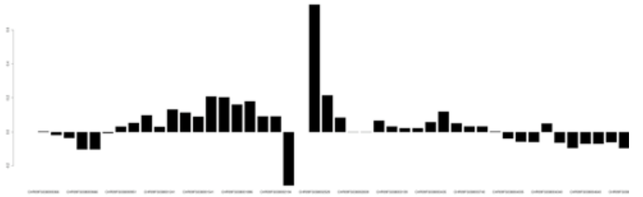

Phox2a

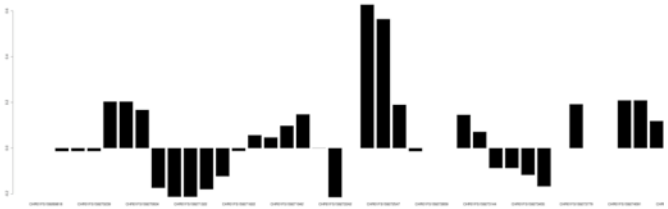

Rag1

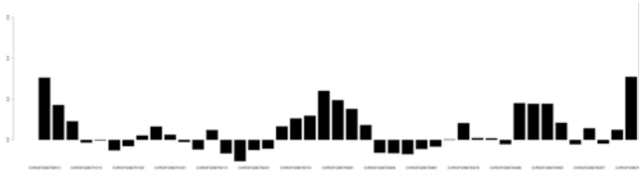

Rai14

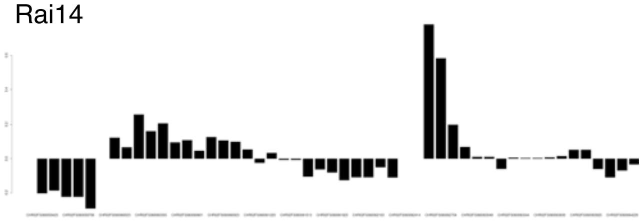

RGD1303127

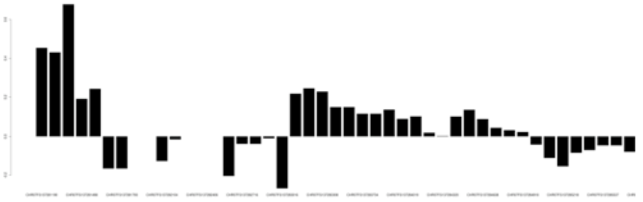

RGD1305721

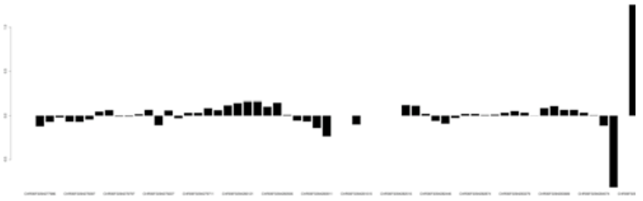

RGD1307325

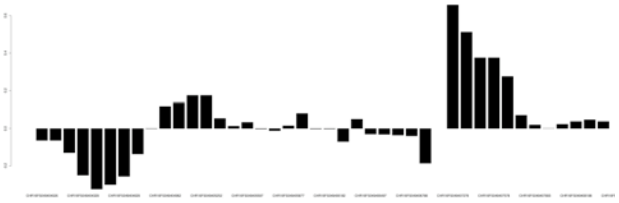

RGD1560888

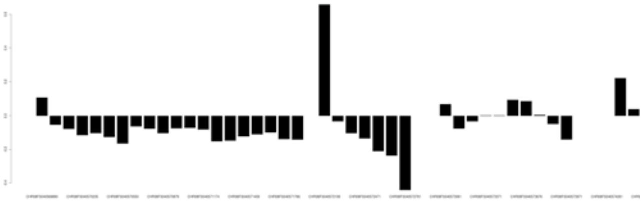

RGD1562533

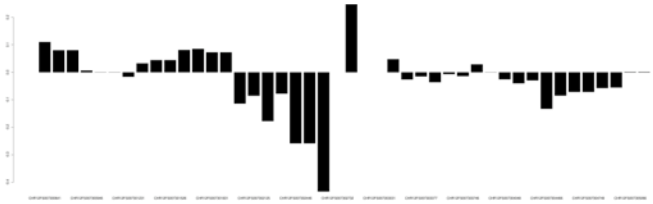

RGD1562638

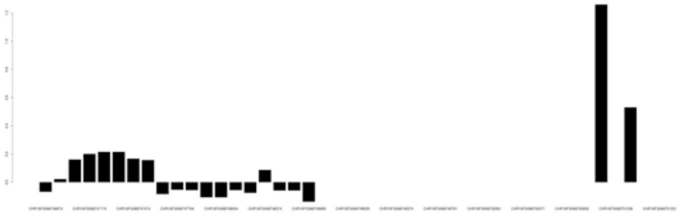

RGD1565947

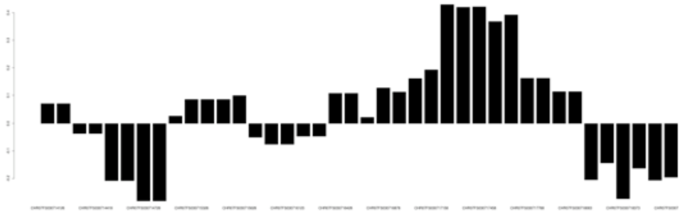

Rpl24

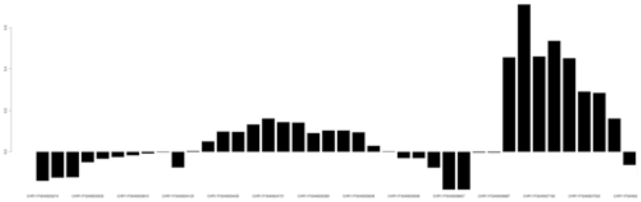

Rtf1

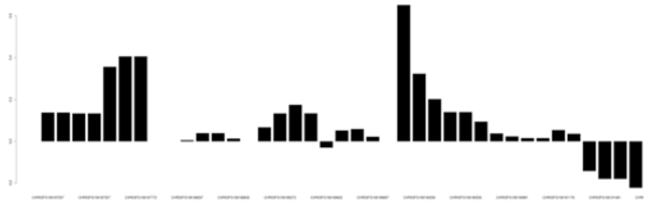

Sar1b

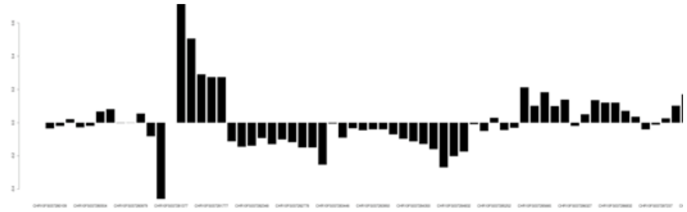

Sdha

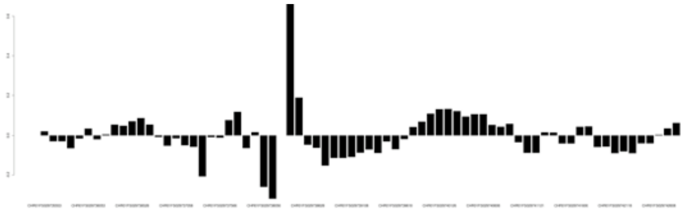

Sdhd

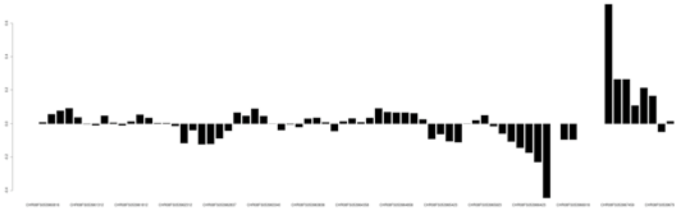

Sec1

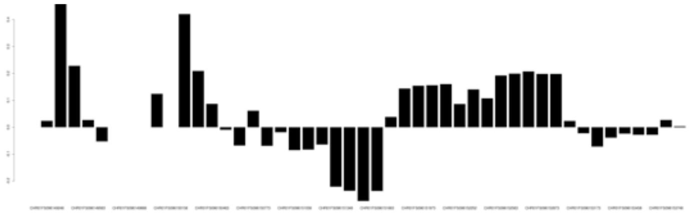

Sec24a

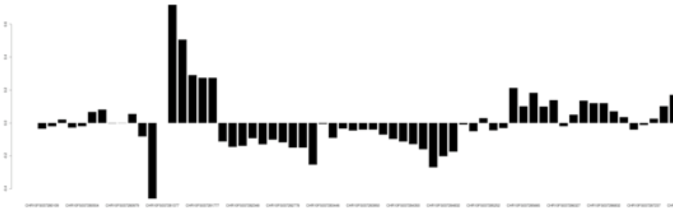

Tex10

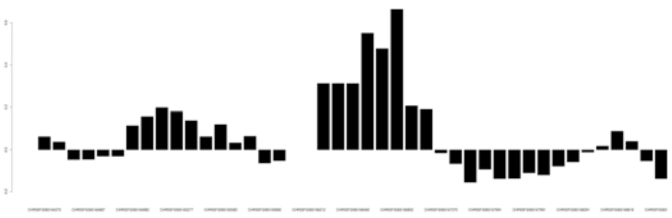

Timm8b

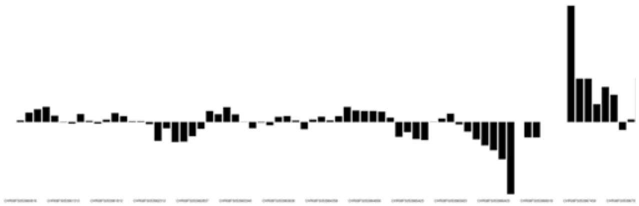

Tmed4

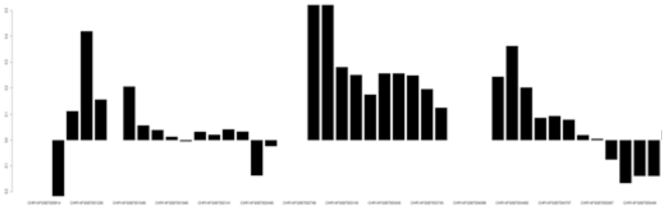

Tmprss6

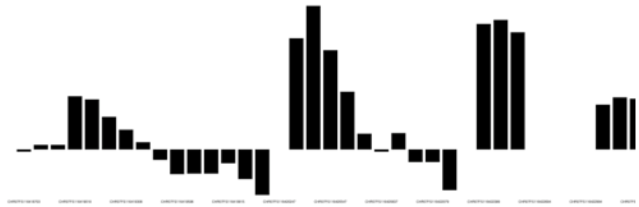

Tpi1

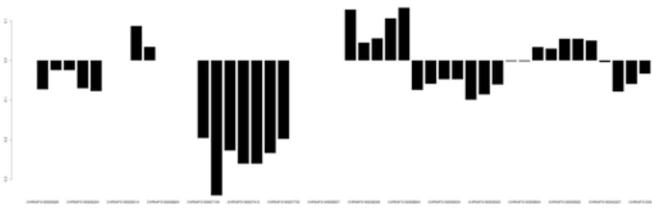

Tsen2

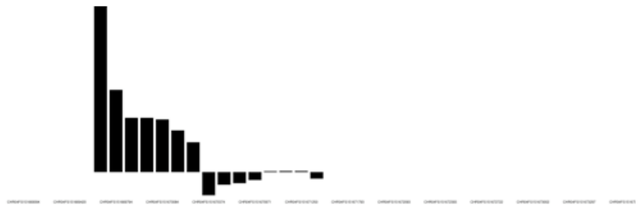

Vom1r59

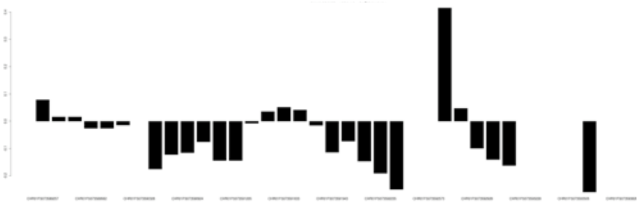

Vom2r8

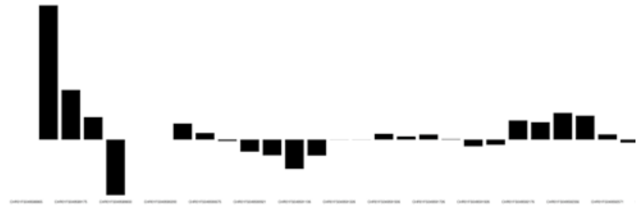

Vom2r11

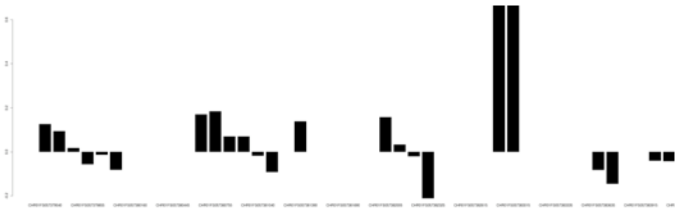

Zfp354a

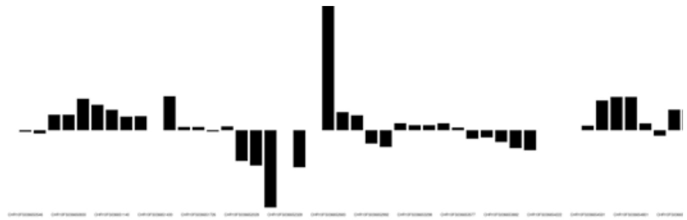

Znf507

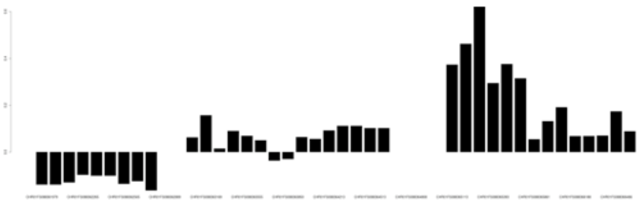

Supplement: Figure S1 — SRY downstream direct binding target gene promoters. The positive hybridization is specific to SRY ChIP-DNA signal and negative hybridization to the non-immune IgG ChIP-DNA signal. Hybridization signals are the average of three biological replicates of ChIP assays. Hybridization signals below the statistical significance of p<1×10^−7^ was not considered. Data represent assays from three different experiments and biological replicates. (PDF) [file pone.0043380.s001.pdf]
