# Supplementary figures and images for: Global Genome Analysis of the Downstream Binding Targets of Testis Determining Factor SRY and SOX9
Source: PLoS One. 2012 Sep 12;7(9):e43380. doi: 10.1371/journal.pone.0043380 (PMC3440412; doi:10.1371/journal.pone.0043380)

## Supplemental Figure S2

Hybridization signals masked by adjacent larger peaks of IgG binding

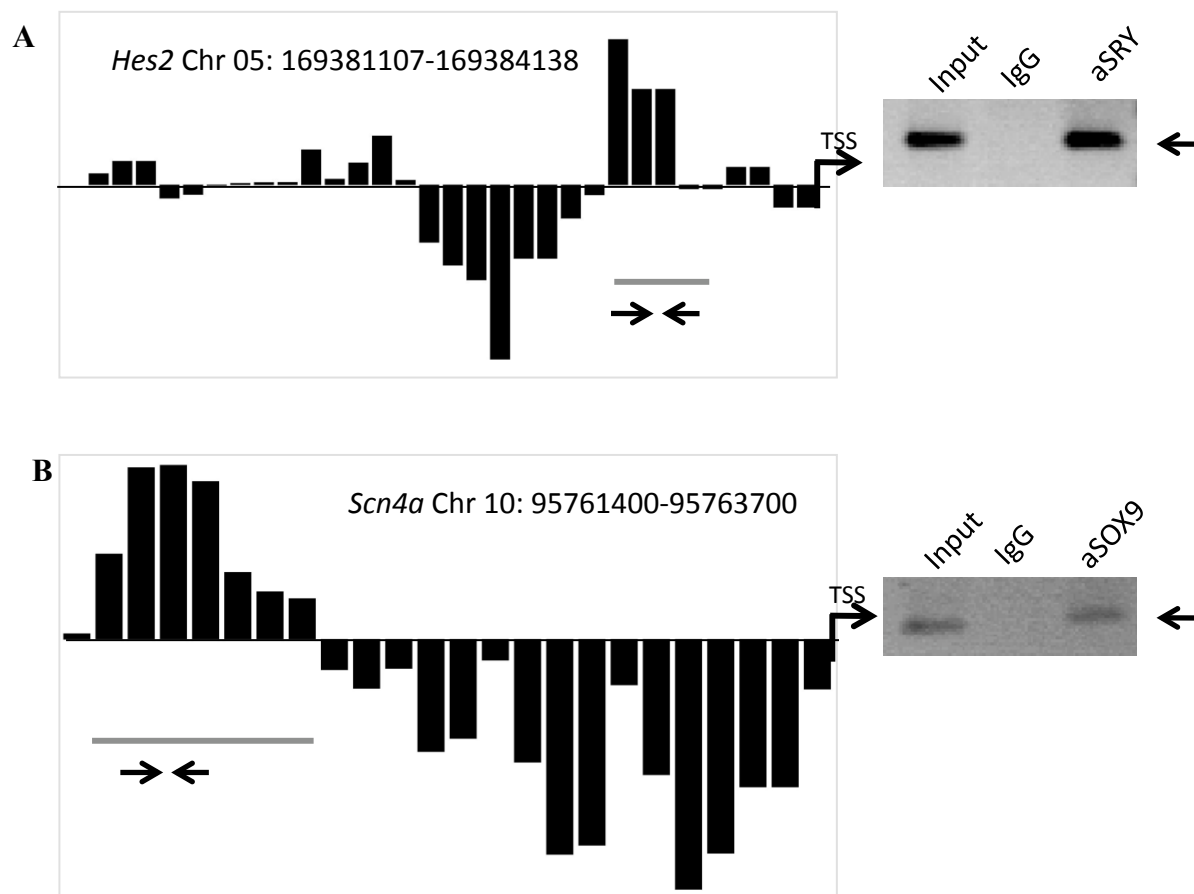

Supplement: Figure S2 — Hybridization signals masked by adjacent larger peaks of IgG binding. (A) Hes2 hybridization profile with horizontal bar with arrows identifying SRY binding site and PCR confirmation of SRY with anti SRY (aSRY), IgG and input DNA. (B) Scn4a hybridization profile with horizontal bar for SOX9 binding and ChIP-PCR confirmation of Sox9 (aSOX9). ChIP DNA from IgG represented negative control (IgG) in PCR. PCR was conducted on 200 ng DNA amplified by whole genome amplification kit (Sigma). Data represent ChIP-PCR assay from three different experiments and biological replicates. (PDF) [file pone.0043380.s002.pdf]

### Supplemental Figure S3

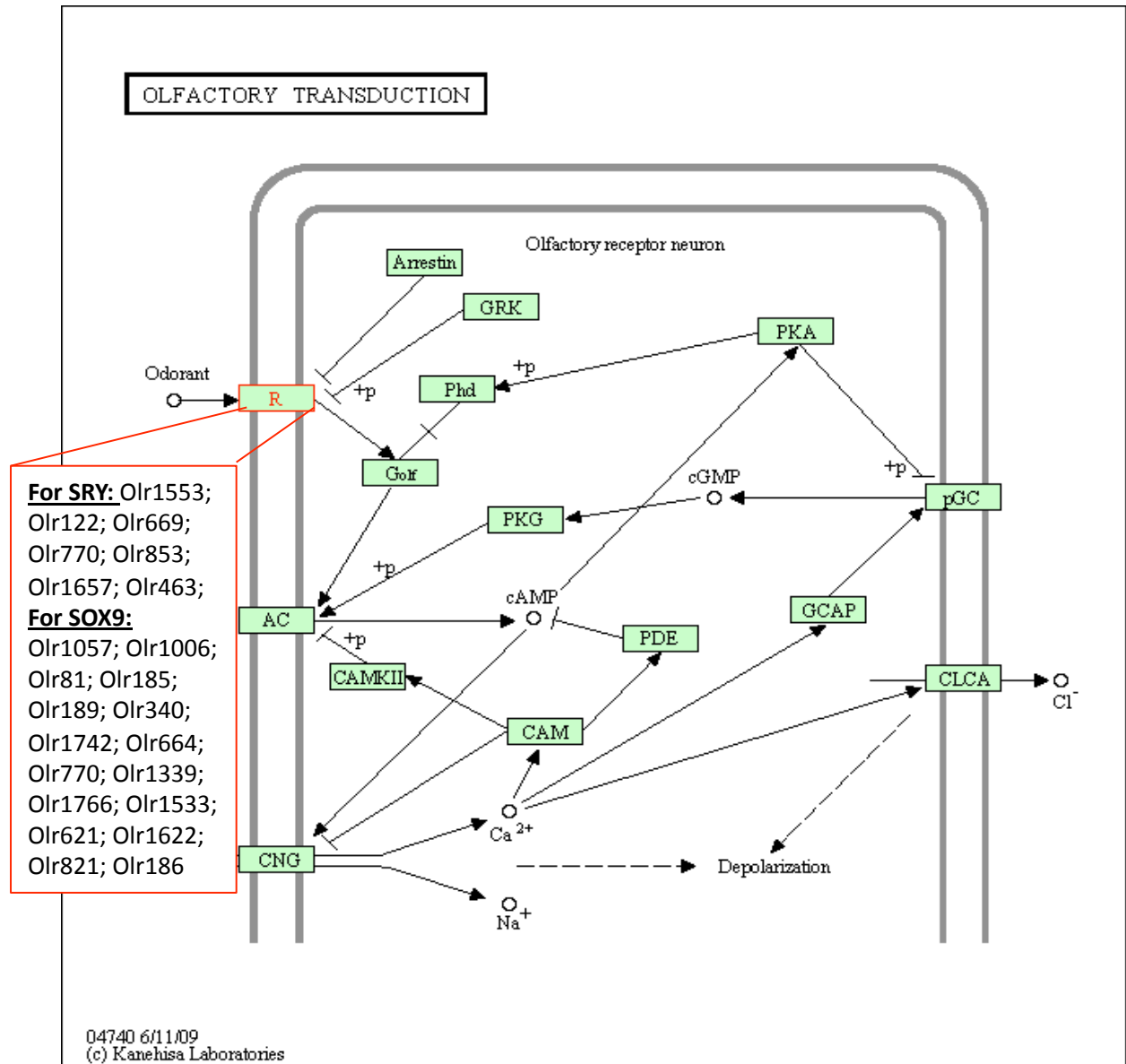

Supplement: Figure S3 — Olfactory transduction signaling pathway from KEGG pathway. The SRY and SOX9 direct binding targets are listed in the olfactory receptor insert box. (PDF) [file pone.0043380.s003.pdf]
