# Supplementary material for: Global Genome Analysis of the Downstream Binding Targets of Testis Determining Factor SRY and SOX9
Source: PLoS One. 2012 Sep 12;7(9):e43380. doi: 10.1371/journal.pone.0043380 (PMC3440412; doi:10.1371/journal.pone.0043380)
Supplement: Table S1 — Atypical downstream binding targets of SRY during male sex determination in the rat. Atypical targets were pulled down by SRY antibody, but the hybridization occurred through indirect binding as the peak of hybridization signal did not contain the SRY consensus motif. (PDF) [file pone.0043380.s004.pdf]

Supplemental Table S1: Atypical binding targets of SRY during male sex determination

Gene Symbol	GenBank/ Reference Sequense	Associated Region Chromosomal Location	p-value	Gene Title
Abca2	NM_024396	chr3:3596064-3596664	6.84E-19	ATP-binding cassette, member A2
Actn4	NM_031675	chr1:84072540-84073231	9.73E-09	Actinin alpha 4
Actr1b	NM_001039028	chr9:35640954-35641659	2.49E-20	ARP1 actin-related protein 1 homolog B
Adam23	NM_001029899	chr9:62105528-62106243	7.21E-10	ADAM metallopeptidase domain 23
Adap2	NM_020101	chr10:68359207-68360102	2.38E-09	ArfGAP with dual PH domains 2
Adcy3	NM_130779	chr6:27118846-27119446	2.90E-10	Adenylate cyclase 3
Add1	NM_016990	chr14:81812496-81813096	2.29E-10	Adducin 1 (alpha)
Agpat1	NM_212458	chr20:4248161-4249165	2.09E-13	1-acylglycerol-3-phosphate O-acyltransferase 1
Akp3	NM_022680	chr9:85922659-85923259	7.40E-11	Alkaline phosphatase 3, intestine
Amigo1	NM_206881	chr2:203752199-203752879	1.84E-15	Adhesion molecule with Ig like domain 1
Anp32a	NM_012903	chr8:66488052-66488652	7.24E-14	Acidic nuclear phosphoprotein 32 A
Aox3l1	NM_001008522	chr9:56944915-56945610	3.89E-09	Aldehyde oxidase 3-like 1
Apoc1	NM_012824	chr1:78999135-79000038	1.57E-24	Apolipoprotein C-I
Arhgap9	NM_001080789	chr7:67279363-67279963	6.21E-09	Rho GTPase activating protein 9
Arl9	NM_212459	chr14:33459931-33460990	5.34E-26	ADP-ribosylation factor-like 9
Arntl	NM_024362	chr1:171058033-171058739	3.81E-15	Aryl hydrocarbon receptor nuclear translocator-like
Atf6b	NM_001002809	chr20:4198783-4199383	2.95E-21	Activating transcription factor 6 beta
Avpr1b	NM_017205	chr13:44537567-44538247	7.64E-09	Arginine vasopressin receptor 1B
Axin1	NM_024405	chr10:15408241-15409121	1.73E-09	Axin 1
Bak1	NM_053812	chr20:5266081-5266681	3.04E-11	BCL2-antagonist/killer 1
Blvrb	NM_001106236	chr1:82527879-82528479	3.31E-08	Biliverdin reductase B (flavin reductase (NADPH))
Bola1	NM_001071776	chr2:191006717-191007417	2.46E-11	BolA homolog 1 (E. coli)
Bpil3	NM_001107791	chr3:144289166-144289866	1.87E-13	Bactericidal/permeability-increasing protein-like 3
Brd9	NM_001107453	chr1:30162343-30163033	3.54E-13	Bromodomain containing 9
Capn7	NM_001030037	chr16:6780437-6781237	1.95E-16	Calpain 7
Cbln4	NM_001109210	chr3:162920485-162921468	6.82E-16	Cerebillin 4 precursor gene
Ccdc17	NM_001014067	chr5:136880009-136880700	6.72E-09	Coiled-coil domain containing 17
Cd163	NM_001107887	chr4:160391577-160392272	1.60E-09	CD163 molecule
Cd7	NM_001107074	chr10:110251934-110252735	1.49E-14	Cd7 molecule
Cd74	NM_013069	chr18:56753899-56754499	7.92E-08	Cd74 molecule, histocompatibility complex
Cdc20	NM_171993	chr5:138916314-138917120	5.18E-09	Cell division cycle 20 homolog (S. cerevisiae)
Cdx2	NM_023963	chr12:8294748-8295348	3.57E-10	Caudal type homeo box 2
Cmtm1	NM_001029914	chr19:618223-619113	2.08E-13	CKLF-like MARVEL transmembrane 1
Cnga3	NM_053495	chr9:36176305-36177014	1.10E-08	Cyclic nucleotide gated channel alpha 3
Cobra1	NM_001107817	chr3:3376385-3376985	4.21E-08	Cofactor of BRCA1
Crip	NM_001134933	chr6:138159509-138160109	1.07E-17	Cysteine-rich intestinal protein
Cstf3	NM_001077672	chr3:89919199-89919799	1.06E-11	Cleavage stimulation factor, subunit 3
Cyp11a1	NM_017286	chr8:61791334-61792086	5.71E-16	Cytochrome P450, family 11 subfamily a1
Cyp2d5	NM_173304	chr7:120765242-120765842	9.04E-08	Cytochrome P450, family 2, subfamily d5
Cyp3a23/3a1	NM_013105	chr12:9598130-9598730	2.49E-11	cytochrome P450, family 3, subfamily a, polypeptide 23
Dapk3	NM_022546	chr7:10019422-10020349	5.78E-10	Death-associated protein kinase 3
Dhodh	NM_001008553	chr19:39476737-39477337	6.95E-29	Dihydroorotate dehydrogenase
Dpm3	NM_001109331	chr2:181442815-181443510	1.99E-08	Dolichyl-phosphate mannosyltransferase 3
Eef2	NM_017245	chr7:10019422-10020349	5.78E-10	Eukaryotic translation elongation factor 2
Enoph1	NM_001009391	chr14:10859242-10859842	2.09E-23	Enolase-phosphatase 1
Fam110b	BC091289	chr5:19260558-19261257	3.26E-08	Family with sequence similarity 110, member B
Fam166a	BC097396	chr3:3376385-3376985	4.21E-08	Family with sequence similarity 166, member A
Fam3b	NM_001107102	chr11:37508106-37508706	2.19E-08	Family with sequence similarity 3, member B
Fdxr	NM_024153	chr10:105350453-105351348	6.10E-10	Ferredoxin reductase
Fetub	NM_053348	chr11:80284316-80285011	1.50E-08	Fetuin B
Fkbpl	NM_001002818	chr20:4198783-4199383	2.95E-21	FK506 binding protein-like
Flrt2	NM_001106750	chr6:119572214-119573101	7.78E-11	Fibronectin leucine rich transmembrane protein 2

Ggnbp1	NM_001009972	chr20:5266081-5266681	3.04E-11	Gametogenetin-binding protein 1
Gif	NM_017162	chr1:214519818-214520603	1.25E-08	Gastric intrinsic factor
Gipc2	NC_005101	chr2:250647056-250647748	3.19E-10	GIPC PDZ domain containing family, member 2
Gjb4	NM_053984	chr5:146802870-146803763	1.26E-08	Gap junction protein, beta 4
Gjb5	NM_019241	chr5:146802870-146803763	1.26E-08	Gap junction protein, beta 5
Grinl1a	NM_183402	chr8:75957303-75957903	9.91E-08	Glutamate receptor, ionotropic
Grip2	NM_138535	chr4:126001153-126001753	1.05E-11	Glutamate receptor interacting protein 2
Gstm3	NM_020540	chr2:203539392-203540095	2.33E-09	Glutathione S-transferase mu 3
Gtf2e1	NM_001100556	chr11:64970173-64970773	6.55E-16	General transcription factor IIE, 1 alpha
Hal	NM_017159	chr7:30519619-30520431	8.75E-10	histidine ammonia lyase
Hirip3	NM_001025725	chr1:186042526-186043515	4.41E-08	HIRA interacting protein 3
Hmga1	NM_139327	chr20:5768865-5769565	1.50E-19	High mobility group AT-hook 1
Hnrpdl	NM_001033696	chr14:10859242-10859842	2.09E-23	Heterogeneous nuclear ribonucleoprotein D-like
Id2	NM_013060	chr6:42785587-42786187	1.49E-10	Inhibitor of DNA binding 2
Ino80e	NM_001013900	chr1:186042526-186043515	4.41E-08	INO80 complex subunit E
Insl3	NM_053680	chr16:18886728-18887515	8.12E-08	Insulin-like 3
Itga5	NM_001108118	chr7:142276221-142276821	3.92E-17	Integrin, alpha 5
Katnb1	NM_001024746	chr19:10373728-10374438	6.09E-08	Katanin p80 subunit B 1
Kcnc3	NM_053997	chr1:95064454-95065159	5.52E-09	Potassium voltage gated channel, member 3
Kcnh6	NM_053937	chr10:95401433-95402033	5.75E-09	Potassium voltage-gated channel, H 6
Kcnj11	NM_031358	chr1:96618173-96618773	7.03E-08	Potassium channel, subfamily J, member 11
Kcns1	NM_053954	chr3:155112328-155113005	4.04E-10	Potassium voltage-gated channel, S1
Kctd3	NM_001107199	chr13:105158630-105159230	1.11E-08	Potassium channel tetramerisation domain 3
Krt14	NM_001008751	chr10:89147890-89148490	5.58E-09	Keratin 14
Krt8	NM_199370	chr7:140723237-140723837	1.23E-09	Keratin 8
Lix1l	NM_001024303	chr2:191399971-191400571	5.65E-09	Lix1 homolog (mouse)-like
LOC10023317	NM_001142941	chr6:127030974-127031659	2.10E-08	Hypothetical protein LOC100233176
LOC259246	NM_147214	chr5:78712384-78712984	7.80E-09	Alpha-2u globulin PGCL1
LOC298116	M26836	chr5:78712384-78712984	7.80E-09	Hypthetical protein LOC298116
LOC498155	NM_001047924	chr12:16295287-16296182	1.34E-11	Hypthetical protein LOC498155
LOC680531	BC158701	chr3:3534830-3535430	2.74E-15	Similar to CG3880-PA
LOC684097	NM_001044283	chr20:2644863-2645763	8.06E-09	Similar to sorting nexin 3
LOC687707	NC_005109	chr10:57495548-57496148	9.08E-08	Hypothetical protein LOC687707
Lrrc14	NM_001024354	chr7:114759355-114759955	2.11E-08	Leucine rich repeat containing 14
Lta	NM_080769	chr20:3661436-3662139	1.29E-09	Lymphotoxin alpha (TNF superfamily, member 1)
Ly6al	NM_001128099	chr7:113437714-113438314	4.50E-30	Lymphocyte antigen 6 complex, locus A-like
Ly6g6d	NM_001001970	chr20:3809455-3810400	5.12E-08	Lymphocyte antigen 6 complex, locus G6D
Ly6g6e	NM_001001972	chr20:3809455-3810400	5.12E-08	Lymphocyte antigen 6 complex, locus G6E
Max	NM_022210	chr6:99553826-99554426	4.55E-09	MYC associated factor X
Mettl14	NM_001106470	chr2:220116649-220117359	2.77E-13	Methyltransferase like 14
Mill2	NM_001017468	chr1:78199499-78200193	1.38E-08	MHC I like leukocyte 2
Mpzl1	NM_001007728	chr13:81357787-81358485	6.69E-19	Myelin protein zero-like 1
Mthfd1	NM_022508	chr6:98849388-98850083	7.06E-09	Methylenetetrahydrofolate dehydrogenase 1
Nat8b	NM_133558	chr4:120082762-120083460	1.99E-08	N-acetyltransferase 8B
Necab3	NM_001098724	chr3:145034073-145034673	1.12E-08	N-terminal EF-hand calcium binding protein 3
Neurl	NM_001107605	chr1:252502203-252502803	3.23E-13	Neuralized homolog
Nmur1	NM_023100	chr9:85149185-85150163	2.49E-08	Neuromedin U receptor 1
Nr4a3	NM_031628	chr5:64713331-64713931	4.09E-08	Nuclear receptor subfamily 4, group A, member 3
Nsg1	NM_024128	chr14:77929546-77930251	2.24E-11	Neuron specific gene family member 1
Olfml2b	NM_001107195	chr13:86478204-86478804	7.33E-12	Olfactomedin-like 2B
Olr1631	NM_001000837	chr15:26595553-26596254	2.46E-11	Olfactory receptor 631
Onecut1	NM_022671	chr8:79644710-79645500	7.65E-12	One cut homeobox 1
Panx1	NM_199397	chr8:11852319-11852919	2.51E-09	Pannexin 1
Pbk	NM_001079937	chr15:45225632-45226232	9.94E-08	PDZ binding kinase
Pcdha3	AB113386	chr18:29678015-29678615	1.52E-08	Protocadherin alpha 3
Pde9a	NM_138543	chr20:9812969-9813644	6.10E-21	Phosphodiesterase 9A

Prima1	NM_001108721	chr6:127523563-127524643	3.56E-08	Proline rich membrane anchor 1
Proca1	NM_001025758	chr10:64442921-64443631	1.00E-12	Protein interacting with cyclin A1
Procr	NM_001025733	chr3:146265137-146265737	1.20E-11	Protein C receptor, endothelial
Rab34	NM_001012140	chr10:64442921-64443631	1.00E-12	RAB34, member RAS oncogene family
Rab3c	NM_133536	chr2:41673441-41674135	1.87E-13	RAB3C, member RAS oncogene family
Rabl3	NM_001108319	chr11:64970173-64970773	6.55E-16	RAB, member of RAS oncogene family-like 3
Rag1ap1	NM_001106445	chr2:181442815-181443510	1.99E-08	Secombination activating gene 1 activating protein 1
Rasal3	NM_001134562	chr7:12968996-12969706	1.65E-26	RAS protein activator like 3
Recql4	NM_001130494	chr7:114759355-114759955	2.11E-08	RecQ protein-like 4
RGD1306371	NM_001134558	chr14:83163382-83164077	3.00E-10	RGD 1306371
RGD1306446	NM_001008554	chr14:23195606-23196289	4.06E-11	RGD 1306446
RGD1306595	NM_001025626	chr10:68345133-68345733	6.77E-09	Similar to hypothetical protein
RGD1306839	NM_001106347	chr1:233044361-233044961	2.62E-08	Similar to RIKEN cDNA 5033414D02
RGD1306917	BC169127	chr20:5372754-5373555	1.21E-08	Similar to RIKEN cDNA 2900010M23
RGD1308385	NM_001107785	chr3:133373792-133374392	7.06E-123	Similar to RIKEN cDNA 1700010M22
RGD1560608	NC_005105	chr6:136160610-136161210	5.97E-09	Similar to novel protein
RGD1560927	NM_001109315	chrX:7708999-7709808	1.01E-12	RGD 1560927
RGD1561459	NM_001108493	chr1:154817409-154818009	1.37E-08	Similar to RIKEN cDNA 1810020D17
RGD1562351	NC_005103	chr4:21390571-21391171	2.21E-08	Similar to chromosome 7 open reading frame 23
Rhobtb2	NM_001013133	chr15:50186382-50187077	4.36E-13	Rho-related BTB domain containing 2
Rnf111	NM_001106836	chr8:74982822-74983422	1.16E-10	Ring finger protein 111
Rnf5	NM_001109025	chr20:4248161-4249165	2.09E-13	Ring finger protein 5
Rpl41	NM_139083	chr7:1840367-1841162	2.52E-11	Ribosomal protein L41
Rps24	NM_031112	chr16:40896-41787	4.18E-09	Ribosomal protein S24
Rspo3	NM_001100990	chr1:29071493-29072093	4.59E-12	R-spondin 3 homolog (Xenopus laevis)
RT1-CE12	NM_001008835	chr20_random:144766-145766	5.49E-09	RT1 class I, locus CE12
RT1-CE14	BC083799	chr20_random:144766-145766	5.49E-09	RT1 class I, locus CE14 /// RT1 class I, locus CE13
RT1-CE16	NM_001008839	chr20_random:42708-43385	9.16E-09	RT1 class I, locus CE16
S1pr4	NM_001108075	chr7:9687768-9688453	7.52E-10	Sphingosine-1-phosphate receptor 4
Scn4a	NM_013178	chr10:95763150-95763750	1.73E-10	Sodium channel, voltage-gated, type IV, alpha subunit
Scn5a	NM_013125	chr8:124545886-124546567	1.22E-08	Sodium channel, voltage-gated, type V, alpha subunit
Spata9	NM_001106399	chr2:3011594-3012277	1.66E-08	Spermatogenesis associated 9
Sv2c	NM_031593	chr2:26554389-26554989	2.16E-26	Synaptic vesicle glycoprotein 2c
Tbkbp1	NM_172021	chr10:85869784-85870546	1.75E-14	TBK1 binding protein 1
Thap1	NM_001008340	chr16:70272253-70272953	8.02E-08	THAP domain containing, apoptosis associated protein 1
Tmem119	NM_001107155	chr12:43859955-43860555	4.55E-14	Transmembrane protein 119
Tmem25	NM_001109528	chr8:47754509-47755109	3.07E-08	Transmembrane protein 25
Tnf	NM_012675	chr20:3661436-3662139	1.29E-09	Tumor necrosis factor (TNF superfamily, member 2)
Tomm20	NM_152935	chr19:56865042-56865642	6.91E-12	Translocase of outer mitochondrial membrane 20 homolog (yeast)
Tpra1	NM_053534	chr4:122998126-122998726	1.28E-33	Transmembrane protein, adipocyte asscociated 1
Trip13	NM_001011930	chr1:30162343-30163033	3.54E-13	Thyroid hormone receptor interactor 13
Tspan33	NM_001109227	chr4:56583349-56583949	1.84E-08	Tetraspanin 33
Ttc36	NM_001005546	chr8:47754509-47755109	3.07E-08	Tetratricopeptide repeat domain 36
Ttyh1	NM_001106225	chr1:69482630-69483230	2.99E-09	Tweety homolog 1 (Drosophila)
Uap1l1	NM_001134516	chr3:3534830-3535430	2.74E-15	UDP-N-acteylglucosamine pyrophosphorylase 1-like 1
Ubr7	NM_001007705	chr6:127030974-127031659	2.10E-08	Ubiquitin protein ligase E3 component n-recognin 7
Ucma	NM_001106121	chr17:84391284-84391884	3.22E-08	Upper zone of growth plate and cartilage matrix associated
Ufc1	NM_001003709	chr13:87191595-87192195	3.61E-09	Ubiquitin-fold modifier conjugating enzyme 1
Unc13a	NM_022861	chr12:7556892-7557492	7.33E-12	Unc-13 homolog A (C. elegans)
Uqcrc2	NM_001006970	chr1:179458763-179459498	9.40E-11	Ubiquinol cytochrome c reductase core protein 2
Usp21	NM_001127638	chr13:87180403-87181003	3.15E-09	Ubiquitin specific peptidase 21

Supplemental Table S1. Atypical downstream binding targets of SRY during male sex determination in the rat. Atypical targets were pulled down by SRY antibody, but the hybridization occurred through indirect binding as the peak of hybridization signal did not contain the SRY consensus motif.
