# Supplementary material for: Global Genome Analysis of the Downstream Binding Targets of Testis Determining Factor SRY and SOX9
Source: PLoS One. 2012 Sep 12;7(9):e43380. doi: 10.1371/journal.pone.0043380 (PMC3440412; doi:10.1371/journal.pone.0043380)
Supplement: Table S2 — Questionable downstream binding target genes of SRY. Their hybridization signals were masked by negative binding by IgG, so appeared to be negative in the bioinformatic analysis. These promoters were manually extracted from the database. Confirmation of positive binding in the promoter was done by PCR as shown in the supplemental figure S2. (PDF) [file pone.0043380.s005.pdf]

Supplemental Table S2: Questionable targets of SRY masked by IgG

Gene Symbol	GenBank/ Reference Sequense	Associated Region Chromosomal Location	p-value	Gene Title
Aldh3a1	NM_031972	chr10:47361954-47362554	7.87E-09	Aldehyde dehydrogenase family 3, subfamily A1
Chrna3	NM_052805	chr8:58586553-58587153	1.03E-10	Cholinergic receptor, nicotinic, alpha polypeptide 3
Cyp26a1	NM_130408	chr1:241948077-241948677	1.83E-12	Cytochrome P450, family 26,Polypeptide a1
Dd25	NM_199403	chr13:77575659-77576259	3.39E-08	Hypothetical protein Dd25
Dll3	NM_053666	chr1:83382070-83382670	2.03E-09	Delta-like 3 (Drosophila)
Dtx3l	NM_001109053	chr11:66646344-66646944	1.82E-14	Deltex 3-like (Drosophila)
Exosc3	NM_001107936	chr5:61886253-61887042	1.38E-17	Exosome component 3
F2rl2	NM_053313	chr2:26072501-26073101	5.69E-08	Coagulation factor II (thrombin) receptor-like 2
Gltscr1	NM_001106226	chr1:76321302-76321902	8.23E-11	Glioma tumor suppressor candidate region gene 1
Gpr123	NM_001107559	chr1:199652413-199653313	8.73E-16	G protein-coupled receptor 123
Gpr39	NM_001114392	chr13:38179458-38180058	5.13E-09	G protein-coupled receptor 39
Hemk1	NM_001106853	chr8:112580163-112580848	5.04E-11	HemK methyltransferase family member 1
Hes2	NM_019236	chr5:169384630-169385510	3.05E-10	Hairy and enhancer of split 2 (Drosophila)
Hn1l	NM_001013182	chr10:14213558-14214257	1.45E-08	Hematological and neurological expressed 1-like
Ifi27l2b	NM_206846	chr6:127734901-127735501	3.40E-12	Interferon, alpha-inducible protein 27 like 2B
Kcnn3	NM_019315	chr2:181715456-181716241	1.71E-09	Potassium calcium-activated channel, member N3
Lag3	NM_212513	chr4:161041783-161042573	1.03E-09	Lymphocyte-activation gene 3
Lmna	NM_001002016	chr2:180616936-180617612	4.85E-14	Lamin A
Map1s	NM_001106070	chr16:19029474-19030074	3.84E-11	Microtubule-associated protein 1S
Map2k7	NM_001025425	chr12:1553484-1554177	4.54E-11	Mitogen-activated protein kinase kinase 7
Mpped1	NM_001130569	chr7:121812178-121812778	2.12E-08	Metallophosphoesterase domain containing 1
Ndufs4	NM_001025146	chr2:46480028-46480628	4.99E-08	NADH dehydrogenase Fe-S protein 4
Nxt1	NM_001106521	chr3:137403132-137403732	7.81E-11	NTF2-related export protein 1
Pcgf2	NM_001105836	chr10:86445818-86446713	3.01E-08	Polycomb group ring finger 2
Pdzk1ip1	NM_130401	chr5:135462560-135463357	1.33E-14	PDZK1 interacting protein 1
Pgpep1	NM_201988	chr16:19274841-19275441	8.47E-08	Pyroglutamyl-peptidase I
RGD130716	NM_001025669	chr13:99338319-99338999	3.31E-11	Similar to 0610010K06Rik protein
Sbno1	NM_001107138	chr12:33286676-33287361	4.85E-08	Sno, strawberry notch homolog 1
Scx	NM_001130508	chr7:114503595-114504375	2.75E-10	Scleraxis
Slc30a2	NM_001083122	chr5:153086556-153087647	2.71E-07	Solute carrier family 30, member 2
Slc35a2	NM_001127642	chrX:26650501-26651101	4.81E-10	Solute carrier family 35 , member A2
Tmem95	NM_001134799	chr10:56720709-56721596	5.08E-17	Transmembrane protein 95

Supplemental Table S2. Questionable downstream binding target genes of SRY. Their hybridization signals were masked by negative binding by IgG, so appeared to be negative in the bioinformatic analysis. These promoters were manually extracted from the database. Confirmation of positive binding in the promoter was done by PCR as shown in the supplemental figure S2.
