# Supplementary material for: Global Genome Analysis of the Downstream Binding Targets of Testis Determining Factor SRY and SOX9
Source: PLoS One. 2012 Sep 12;7(9):e43380. doi: 10.1371/journal.pone.0043380 (PMC3440412; doi:10.1371/journal.pone.0043380)
Supplement: Table S3 — Atypical downstream binding targets of SOX9 during male sex determination in the rat. Atypical targets were pulled down by SOX9 antibody, but the hybridization occurred though indirect binding as the peak of hybridization signal did not contain HMG or in vitro derived SOX9 consensus motif. (PDF) [file pone.0043380.s006.pdf]

Supplemental Table S3: Atypical binding targets of SOX9 during male sex determination

Gene Symbol	GenBank/ Reference Sequense	Associated Region Chromosomal Location	p-value	Gene Title
Akap4	NM_024402	chrX:27527026-27527727	3.26E-08	A kinase (PRKA) anchor protein 4
Ceacam10	NM_173339	chr1:80068639-80069239	6.76E-19	Carcinoembryonic antigen cell adhesion molecule 1
Clic2	NM_001009651	chr20:65332-66017	7.22E-10	Chloride intracellular channel 2
Itih4	NM_019369	chr16:6317228-6317970	4.44E-11	Inter alpha-trypsin inhibitor, heavy chain 4
Kazald1	NM_001033064	chr1:250186637-250187318	2.72E-11	Kazal-type serine peptidase inhibitor domain 1
Lgals5	NM_012976	chr10:65078860-65079558	3.65E-24	Lectin, galactose binding, soluble 5
MGC72612	NM_001009538	chr16:20249364-20249964	2.07E-10	Similar to expressed sequence AI449175
Mxd3	NM_145773	chr17:15350263-15350863	6.98E-08	Max dimerization protein 3
Nckap1l	NM_001108119	chr7:142347901-142348583	1.80E-11	NCK associated protein 1 like
Npffr1	NM_022291	chr20:28981461-28982061	2.50E-08	Neuropeptide FF receptor 1
Olr1144	NM_001000876	chr8:17488843-17489443	3.67E-23	Olfactory receptor 1144
Olr1323	NM_001000472	chr8:42763562-42764162	3.22E-08	Olfactory receptor 1323
Olr1619	NM_001000521	chr15:26241987-26242587	3.08E-34	Olfactory receptor 1619
Olr1686	NM_001001373	chr20:417292-417990	7.43E-18	Olfactory receptor 1686
Olr29	NM_001000691	chr1:142823485-142824188	1.57E-08	Olfactory receptor 29
Olr32	NM_001000690	chr1:142874835-142875435	1.49E-08	Olfactory receptor 32
Pou3f3	NM_138837	chr9:41873902-41874717	8.47E-11	POU class 3 homeobox 3
Prelid1	NM_001009636	chr17:15350263-15350863	6.98E-08	PRELI domain containing 1
Rab24	NM_001015023	chr17:15350263-15350863	6.98E-08	RAB24, member RAS oncogene family
RGD15632(	BC167768	chr7:142347901-142348583	1.80E-11	Hypothetical protein LOC500938
Rpl41	NM_139083	chr7:1839966-1841047	1.50E-13	Ribosomal protein L41
Smgb	NM_080775	chr3:144425259-144425859	6.07E-09	Neonatal submandibular gland protein B
Vom1r81	AY510288	chr4:86727924-86728524	2.37E-09	vomeronasal 1 receptor, 81
Xylt2	NM_022296	chr10:83313447-83314148	2.10E-14	Xylosyltransferase II

Supplemental Table S3. Atypical downstream bining targets of SOX9 during male sex determination in the rat. Atypical targets were pulled down by SOX9 antibody, but the hybridization occurred though indirect binding as the peak of hybridization signal did not contain HMG or in vitro derived SOX9 consensus motif.
