# Supplementary material for: Global Genome Analysis of the Downstream Binding Targets of Testis Determining Factor SRY and SOX9
Source: PLoS One. 2012 Sep 12;7(9):e43380. doi: 10.1371/journal.pone.0043380 (PMC3440412; doi:10.1371/journal.pone.0043380)
Supplement: Table S4 — Questionable downstream targets of SOX9 during male sex determination in the rat. Their hybridization signals were masked by negative binding by IgG, so appeared to be negative in the bioinformatic analysis. These promoters were manually extracted from the database. (PDF) [file pone.0043380.s007.pdf]

Supplemental Table S4: Questionable targets of SOX9 masked by IgG

Gene Symbol	GenBank/ Reference Sequense	Associated Region Chromosomal Location	p-value	Gene Title
Ankrd13a	NM_001012148	chr12:43113572-43114172	6.68E-16	Ankyrin repeat domain 13a
Asmt	NM_144759	chr12:16816154-16816847	2.21E-13	Acetylserotonin O-methyltransferase
Atf6b	NM_001002809	chr20:4198783-4199498	3.47E-10	Activating transcription factor 6 beta
Carkd	NM_001108402	chr16:82858818-82859418	8.30E-08	Carbohydrate kinase domain containing
Cfi	NM_024157	chr2:227277504-227278104	3.17E-08	Complement factor I
Cryl1	NM_175757	chr15:36444127-36444727	1.81E-09	Crystallin, lambda 1
Cyp39a1	NM_001106893	chr9:13005620-13006500	2.49E-17	Cytochrome P450, family 39 polypeptide a1
Ddc	NM_012545	chr14:92791577-92792391	2.25E-08	Dopa decarboxylase
Dpf1	NM_001105729	chr1:84442299-84443102	3.78E-12	D4, zinc and double PHD fingers family 1
Fkbpl	NM_001002818	chr20:4198783-4199498	3.47E-10	FK506 binding protein-like
Git2	NM_001005553	chr12:43113572-43114172	6.68E-16	G protein-coupled receptor kinase 2
Higd2a	NM_001106102	chr17:16084041-16084831	1.45E-11	HIG1 hypoxia inducible, member 2A
Mars	NM_001127659	chr7:67271631-67272426	2.87E-13	Methionine-tRNA synthetase
Mcm7	NM_001004203	chr12:17611755-17612455	2.37E-10	Minichromosome maintenance complex 7
Med13	NM_001107035	chr10:74585486-74586086	9.24E-08	Mediator complex subunit 13
Mrp63	NM_001109649	chr15:36870464-36871160	2.54E-12	Mitochondrial ribosomal protein 63
Mup4	NM_198784	chr5:78222725-78223325	1.84E-14	Major urinary protein 4
Nop16	NM_001047095	chr17:16084041-16084831	1.45E-11	NOP16 nucleolar protein homolog (yeast)
Npsr1	NM_001106808	chr8:23734300-23735000	5.61E-08	Neuropeptide S receptor 1
Olr623	NM_001000651	chr3:72149583-72150555	6.20E-12	Olfactory receptor 623
Osr1	NM_001106716	chr6:33074044-33074935	2.16E-08	Odd-skipped related 1
Pask	NM_001009362	chr9:92619977-92620762	4.08E-10	PAS domain containing serine/threonine kinase
Ppp1r7	NM_001009825	chr9:92619977-92620762	4.08E-10	Protein phosphatase 1, subunit 7
Ppp6c	NM_133589	chr3:18998269-18998869	5.61E-08	Protein phosphatase 6, catalytic subunit
Rap1a	NM_001005765	chr2:201060366-201061471	1.48E-18	RAP1A, member of RAS oncogene family
Rap2a	NM_053741	chr15:105570224-10557122	1.96E-09	RAS related protein 2a
Rbbp9	NM_019219	chr3:133099831-133100431	2.20E-13	Retinoblastoma binding protein 9
Rbm22	NM_001025676	chr18:56506512-56507112	4.33E-08	RNA binding motif protein 22
RGD131068	NM_001008360	chr10:10889187-10889992	3.70E-09	Similar to chromosome 16 ORF 5
Rnft2	NM_001107144	chr12:39458321-39459016	2.06E-27	Ring finger protein, transmembrane 2
Rpl7	NM_001100534	chr5:2316955-2317555	2.18E-09	Ribosomal protein L7
Rps21	NM_031111	chr3:169311014-169311614	2.30E-10	ribosomal protein S21
RT1-CE2	NM_001008840	chr20:3580310-3581116	1.03E-14	RT1 class I, locus CE2
Scn4a	NM_013178	chr10:95762740-95763950	1.79E-12	sodium channel, type IV, alpha subunit
Ska3	NM_001108379	chr15:36870464-36871160	2.54E-12	Spindle and kinetochore associated 3
Slc45a2	NM_001107653	chr2:60349054-60349654	1.61E-08	Solute carrier family 45, member A2
Timm8a1	NM_053370	chr8:112005351-112006051	2.12E-20	Translocase of inner mitochondrial membrane 8 a1
Uba52	NM_031687	chr16:19425824-19426424	4.76E-08	Ubiquitin A-52 residue ribosomal protein fusion 1
Znf655	NM_001008362	chr12:9688661-9689368	1.50E-08	Zinc finger protein 655

Supplemental Table S4. Questionable downstream targets of SOX9 during male sex determination in the rat. Their hybridization signals were masked by negative binding by IgG, so appeared to be negative in the bioinformatic analysis. These promoters were manually extracted from the database.
