# Supplementary material for: Global Genome Analysis of the Downstream Binding Targets of Testis Determining Factor SRY and SOX9
Source: PLoS One. 2012 Sep 12;7(9):e43380. doi: 10.1371/journal.pone.0043380 (PMC3440412; doi:10.1371/journal.pone.0043380)
Supplement: Table S5 — PCR Primers Utilized. (PDF) [file pone.0043380.s008.pdf]

Supplemental Table S5. PCR Primers Utilized

Gene Symbol	Forward Primer	Reverse primer
Sry	CGGGATCCATGTCAAGCGCCCCATGAATGCATTTATG	GCGGAATTCACTTTAGCCCTCCGATGAGGCTGATAT
Higd2a	CGGGGGTGGAGGCGCAAAT	CGGATCGCTGCCCAGGGTTT
Atn1	TGGTCCTGGTCACAGGGTTCCC	TGGGAGGAGCATGCTGTAGGCT
Hes2	GAGCTGGAGGAGGCAAGGCC	ACTGACCTCCGCTGTGCGTG
Fxc1	ACAGACATGGGCTACAACTGGTTGC	CCTGGGTCTCCACCTTCCTGGT
Enam	GTGCAATGTCTCATCTCGGGCCAT	GGCAGCCTGACACTGATGGCTG
Slc22a7	TAGCGCTCGTGACCTCTGCC	CACCCTCCACGGCCTCTGGAT
Tcf21	TGTGAACTGGAAAAGGCCCTGGAA	TCTGAGCAACATACCACACAGGACA
Scn4a	AGCACCTCCGTGGACAGAGCT	TGGTCACGCAGAGGGAGCTG
TESCO	TAATACAGAGGCACTTTAAAGGAATGT	TGGGAGAGAGGATTTGTACCTTTCTTA
